# Molecular Docking, Computational, and Antithrombotic Studies of Novel 1,3,4-Oxadiazole Derivatives

**DOI:** 10.3390/ijms19113606

**Published:** 2018-11-15

**Authors:** Majda Batool, Affifa Tajammal, Firdous Farhat, Francis Verpoort, Zafar A. K. Khattak, Mehr-un-Nisa, Muhammad Shahid, Hafiz Adnan Ahmad, Munawar Ali Munawar, Muhammad Zia-ur-Rehman, Muhammad Asim Raza Basra

**Affiliations:** 1 Institute of Chemistry, University of the Punjab, New Campus, Lahore 54590, Pakistan; majdabatool12@gmail.com (M.B.); affifa.tajammal@yahoo.com (A.T.); farhatfirdous555@gmail.com (F.F.); anmehrunnisa@gmail.com (M.-u.-N.); mshahidkhan403@gmail.com (M.S.); adnan.ahmad.pu@hotmail.com (H.A.A.); 2 Laboratory of Organometallics, Catalysis and Ordered Materials, State Key Laboratory of Advanced Technology for Material Synthesis and Processing, Wuhan University of Technology, Wuhan 430070, China; Francis.Verpoort@ghent.ac.kr (F.V.); zafarchem_qau@yahoo.com (Z.A.K.K.); 3 Division of Science & Technology, University of Education, Township, Lahore 54770, Pakistan; 4 Key Laboratory of Synthetic and Nature Functional Molecule Chemistry of Ministry of Education, Department of Chemistry & Materials Science, Northwest University, Xi’an 710127, China; 5 Applied Chemistry Research Centre, PCSIR Laboratories Complex, Ferozpur Road, Lahore 54600, Pakistan

**Keywords:** factor Xa (F-Xa), cardiovascular diseases (CD), coronary heart disease (CHD), tissue plasminogen activator (t-PA), urokinase (UK), streptokinase (SK), *N*,*N*-dimethyl formamide (DMF)

## Abstract

A new series of 1,3,4-oxadiazoles derivatives was synthesized, characterized, and evaluated for their in vitro and in vivo anti-thrombotic activity. Compounds (**3a**–**3i**) exhibited significant clot lysis with respect to reference drug streptokinase (30,000 IU), and enhanced clotting time (CT) values (130–342 s) than heparin (110 s). High affinity towards 1NFY with greater docking score was observed for the compounds (**3a**, **3i**, **3e**, **3d**, and **3h**) than the control ligand RPR200095. In addition, impressive inhibitory potential against factor Xa (F-Xa) was observed with higher docking scores (5612–6270) with Atomic Contact Energy (ACE) values (−189.68 to −352.28 kcal/mol) than the control ligand RPR200095 (Docking score 5192; ACE −197.81 kcal/mol). In vitro, in vivo, and in silico results proposed that these newly synthesized compounds might be used as anticoagulant agents.

## 1. Introduction

Nowadays cardiovascular diseases (CD) like coronary heart disease (CHD), atherosclerosis, hypertension, and acute myocardial infarction are the main causes of death in humans. Thrombosis is one of the frequently cause of CD [1]. Almost 20 million people are being affected by thrombotic events worldwide every year. Agents that enhance the fibrinolytic activity and inhibit thrombus formation are important for the treatment and prevention of cerebrovascular and CD [2]. Thrombin (EC.3.4.21.5) is activated and subsequently fibrinogen starts forming fibrin which clots the blood after injury or trauma. Accumulation of fibrin in blood vessels increases thrombosis resulting in various cardiovascular diseases (CVDs) and myocardial infarction [3].

Thrombolysis is the breakdown of blood clots by pharmacological action of drugs and reduces the risk of CVDs [4]. Thrombus disturbs blood flow by hindering the vein, consequently denying the tissues of ordinary blood stream and oxygen supply. Thrombolytic agents such as Urokinase (UK), Streptokinase (SK), and t-PA are frequently used to dissolve the clot in the management of thrombosis [5]. Tissue plasminogen activator (t-PA) enacts the plasmin that digests the fibrin strands supporting the blood clot and restores the normal blood flow to the affected tissues [6]. Their usage is associated with many side effects such as hyper risk of hemorrhage, lack of specificity, and anaphylactic reaction [7]. These drugs have short half-lives (3–20 min) in the body with greater toxicity levels which may cause systemic bleeding [2]. Because of the side-effects and human genetics, new thrombolytic agents are constantly required. Furthermore, heparin and warfarin also used frequently as anticoagulatory agents to prevent the formation of thrombus by inhibiting the factor thrombin, Xa, prothrombin, and X which is present at the junction of intrinsic and extrinsic pathway.

The 1,3,4-oxadizole moiety is a versatile pharmacore for designing potentially biological active agents because of its significant role in medicinal chemistry and wide range of applications as a pharmacological and pharmaceutical agent [8]. These have been found to exhibit various biological activities such as hypoglycemic [9], anti-HIV [10], anticonvulsant [11], antimalarial [12], anti-tubercular [13], analgesic [14], lipid peroxidation inhibition [15], and genotoxic affects [16]. 2,5-Disubstituted-1,3,4-oxadiazol-2-thiol and its derivatives possess antimicrobial, anti-inflammatory, antiviral [11], and anticoagulation activities [17]. Oxadiazole is involved in the inhibition of Factor-Xa (F-Xa), one of the pharmaceutical approaches, and is directly involved in thrombin formation from prothrombin [18,19]. Therefore, the inhibition of F-Xa is considered to be an effective treatment for many clot lysis events with a low risk of bleeding compared to direct thrombin inhibition. Amplified generation of thrombin can be suppressed by F-Xa inhibitor without disturbing the levels necessary to primary homeostasis [20].

Keeping in mind the biological activities of 1,3,4-oxadiazoles, various new derivatives were synthesized and characterized. The in vitro and in vivo antithrombotic efficacy of all derivatives, along with their evaluation by molecular docking and computational studies, were explored. Novel compounds and their inhibitory effect on F-Xa were investigated by molecular docking for visualizing the efficiency of clot lysis agents.

## 2. Results and Discussion

### 2.1. Chemistry

**Scheme 1:**
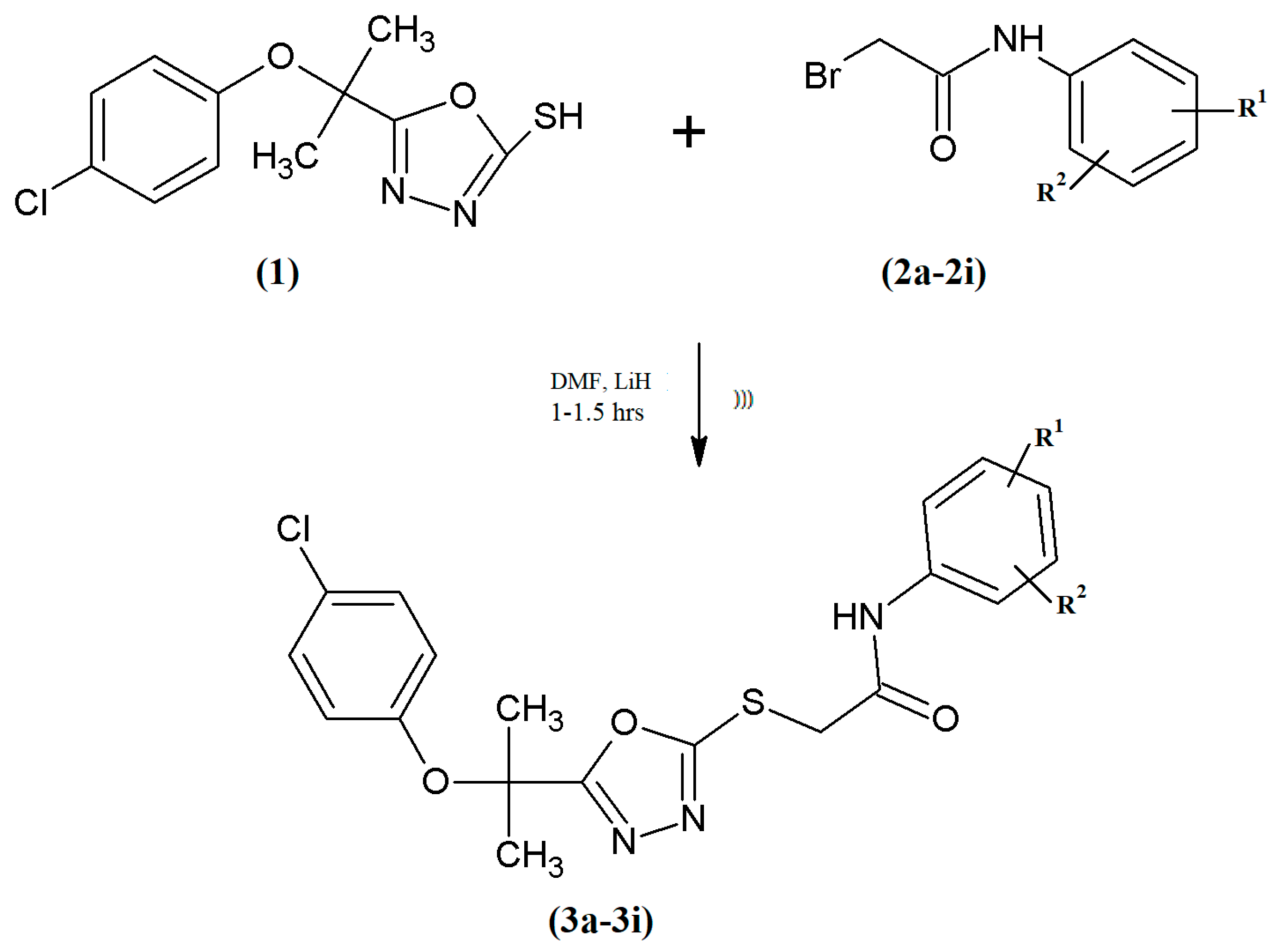
Outline for the synthesis of acetamides (**3a**–**3i**). Reagents & conditions: *N*-substituted-2-bromoacetamide (**2a**–**2i**, one in each case), DMF (*N*,*N*-dimethylformamide), LiH (Lithium hydride), sonication for 1 to 1.5 h at RT (Room temperature), “)))” (Ultrasonic radiations).

The targeted compounds (**3a**–**3i**) (Table 1) were synthesized as depicted in Scheme 1. The key intermediate compounds 5-[2-(4-chlorophenoxy)propan-2-yl]-1,3,4-oxadiazol-2-thiol (**1**) and *N*-substituted-2-bromoacetamides (**2a**–**2i**) were prepared according to a previously reported procedure [21]. The nucleophilic substitution reaction of **1** with **2a**–**2i** was carried out using lithium hydride as a base under ultrasonic radiations at room temperature to obtain the *N*-substituted 5-{[2-(4-chlorophenoxy)propan-2-yl]-1,3,4-oxadiazol-2-ylthio}acetamides (**3a**–**3i**). This modified method gave a better yield in less time than the conventional reported method where only stirring was used to procede the reaction [22]. The supposed structures of targeted compounds were confirmed by spectral data.

The compound **3a** was obtained as white shining white fluffy amorphous flakes. The electron ionization/mass spectroscopy (EI/MS) spectra showed molecular ion peak at *m*/*z* 403 M^+^[C_19_H_19_ClN_3_O_3_S]^+^. The IR spectrum demonstrated the absorption band at 3138 (N–H stretching), 3000 (C–H aromatic stretching), 1671 (C=O amide stretching), and 1552 (C=C aromatic ring stretching). In ^1^H-NMR spectrum the appearance of singlet at δ10.42 represents one proton of NH. In aromatic region, the appearance of two doublets one at δ7.57, *J* = 8 Hz having integration for two protons (H-3′ & H-5′) and the other at δ6.75, *J* = 9 Hz for two protons (H-2′ & H-6′) revealed that aromatic ring is disubstituted at para (1,4) positions. Similarly, the appearance of triplet at δ7.33, *J* = 7.8 Hz for two protons (H-2″ & H-6″), a doublet at δ7.28, *J* = 8.5 Hz for two protons (H-3″ & H-5″), and a triplet at δ7.08, *J* = 7.3 Hz integrate one proton (H-4″) can be assigned to the unsubstituted benzene ring of amide. A singlet at δ4.33 was due to methylene group (CH_2_) linking the amide and sulfanyl group. In aliphatic region of the spectrum a singlet at δ1.71 was assigned to 6H of two methyl groups. Therefore on the basis of above cumulative evidences the structure of **3a** was confirmed as 2-{5-[2-(4-chlorophenoxy)propan-2-yl]-1,3,4-oxadiazol-2-ylthio}-*N*-phenylacetamide. The structures of other *N*-substituted acetamides were also characterized in a similar manner. All the signals in ^1^H-NMR spectrum of (**3a**–**3i**) thoroughly affirmed the successful substitutions on the parent 1,3,4-oxadiazole core.

### 2.2. Antithrombotic Activity

#### 2.2.1. In Vitro Clot Lysis Effect

Results demonstrated that compound **3i** has impressive clot lysis activity (41%) compared to SK (38%). While compound **3c** depicted less clot lysis activity (11%), and the order of the in vitro clot lysis effect of all tested compounds is **3i**>**SK**>**3e**>**3a**>**3d**=**3f**>**3h**>**3g**>**3b**>**1**>**3c**. The results of all synthesized compounds are presented in Table 2.

#### 2.2.2. In Vivo Antithrombotic Activity

Rats were used to evaluate the anticoagulation activity of 1,3,4-oxadiazole derivatives. Clotting time (CT) was used to measure the anticoagulation activity of synthesized compounds. Increasing trend in CT values was found for each compounds (**3a**–**3i**) with increasing the time after their exposure to rats. Heparin was used as reference compound.

**Figure 1:**
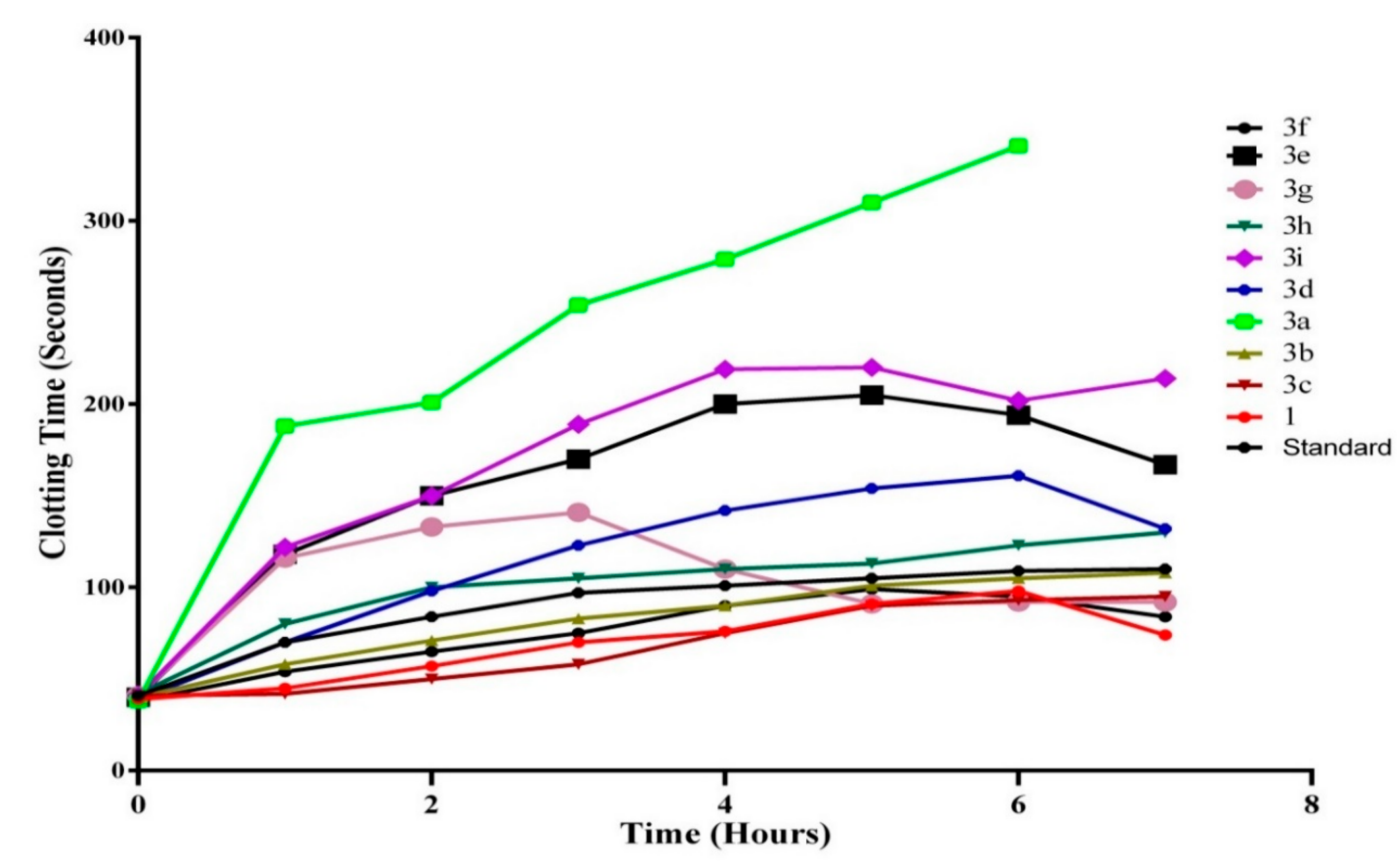
Time course determination of anticoagulation activity of tested compounds **3a**–**3i** and **1**. Heparin was used as standard drug.

All oxadiazole derivatives showed prominent prolongation in clotting time except compound **1**. Compounds **3a** (342 s), **3i** (214 s), **3e** (167 s), **3d** (132 s), and **3h** (130 s) showed CT values greater than heparin (110 s). The order CT values of all the tested oxadiazole derivatives is **3a**>**3i**>**3e**>**3g**>**3d**>**3h**>**3b**>**3f**>**3c**>**1**. The CT results of all tested compounds are presented in Figure 1.

### 2.3. Molecular Docking

The serine protease F-Xa plays a crucial role in blood coagulation process by converting prothrombin to thrombin. This F-Xa is located at the conjunction point of extrinsic and intrinsic pathway. In coagulation process one molecule of F-Xa activates many molecules of prothrombin to thrombin by signal amplification [18,19]. Therefore, the inhibition of F-Xa is considered to be an effective treatment for many thrombotic events with a low risk of bleeding as compared to direct thrombin inhibition [23].

**Figure 2:**
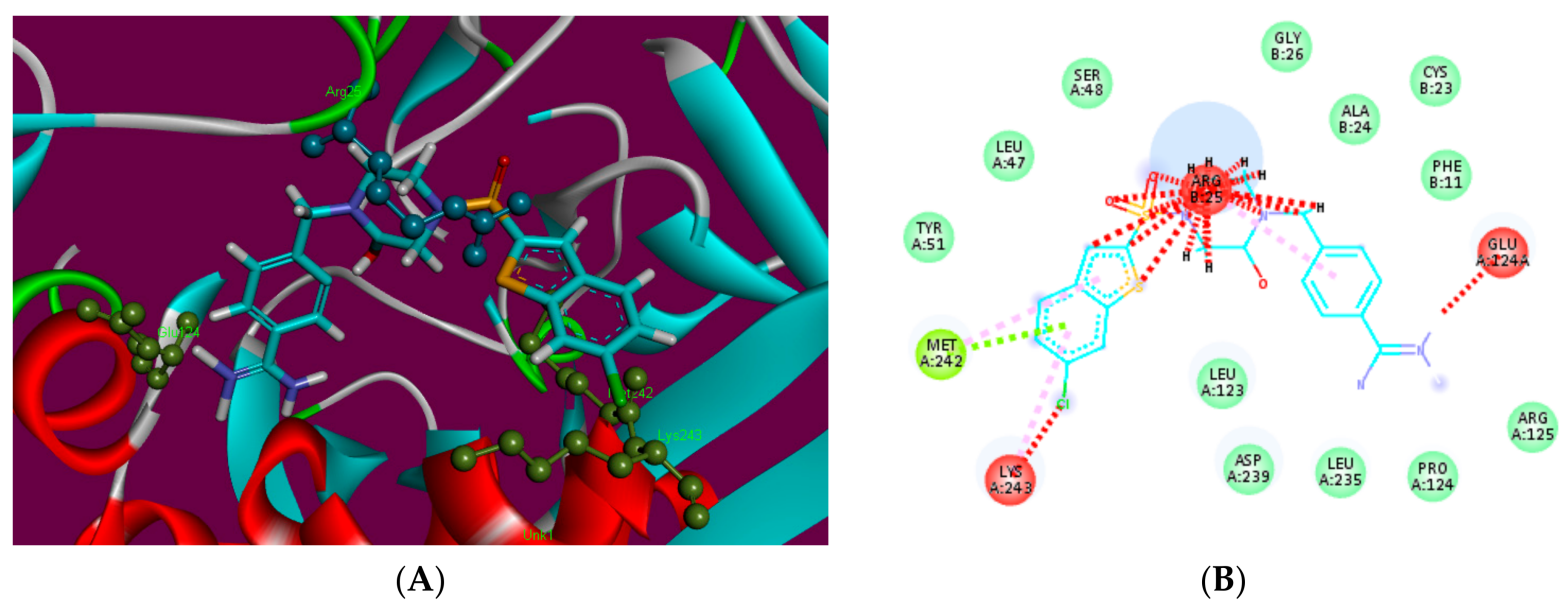
3D (**A**) and 2D (**B**) binding site interactions of standard RPR200095.

**Figure 3:**
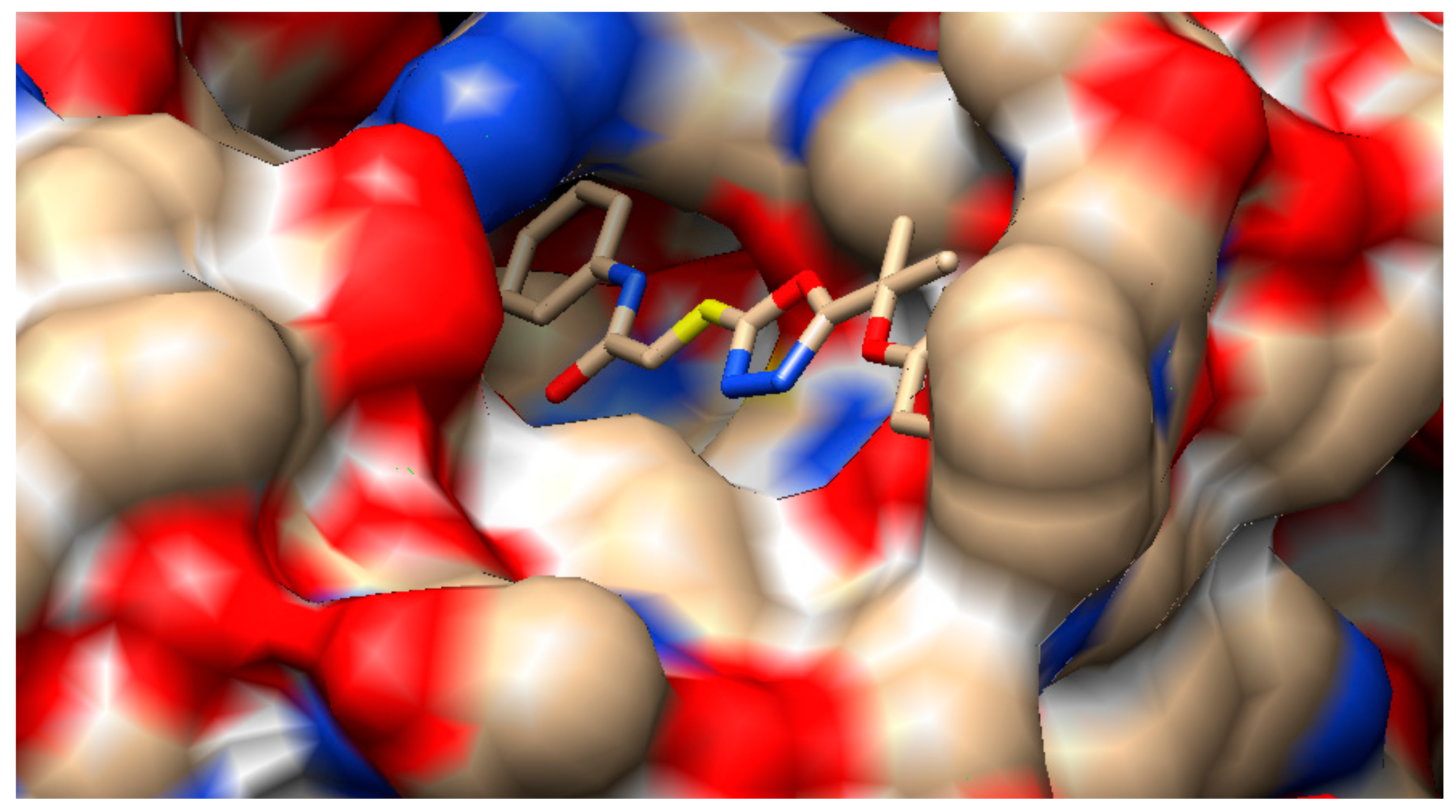
Hyde affinity analysis of most active inhibitor **3a**. The favorably contributing structural elements (atoms and torsions) to the overall binding energy colored blue, unfavorably are colored in red, and neutral elements are in white.

**Figure 4:**
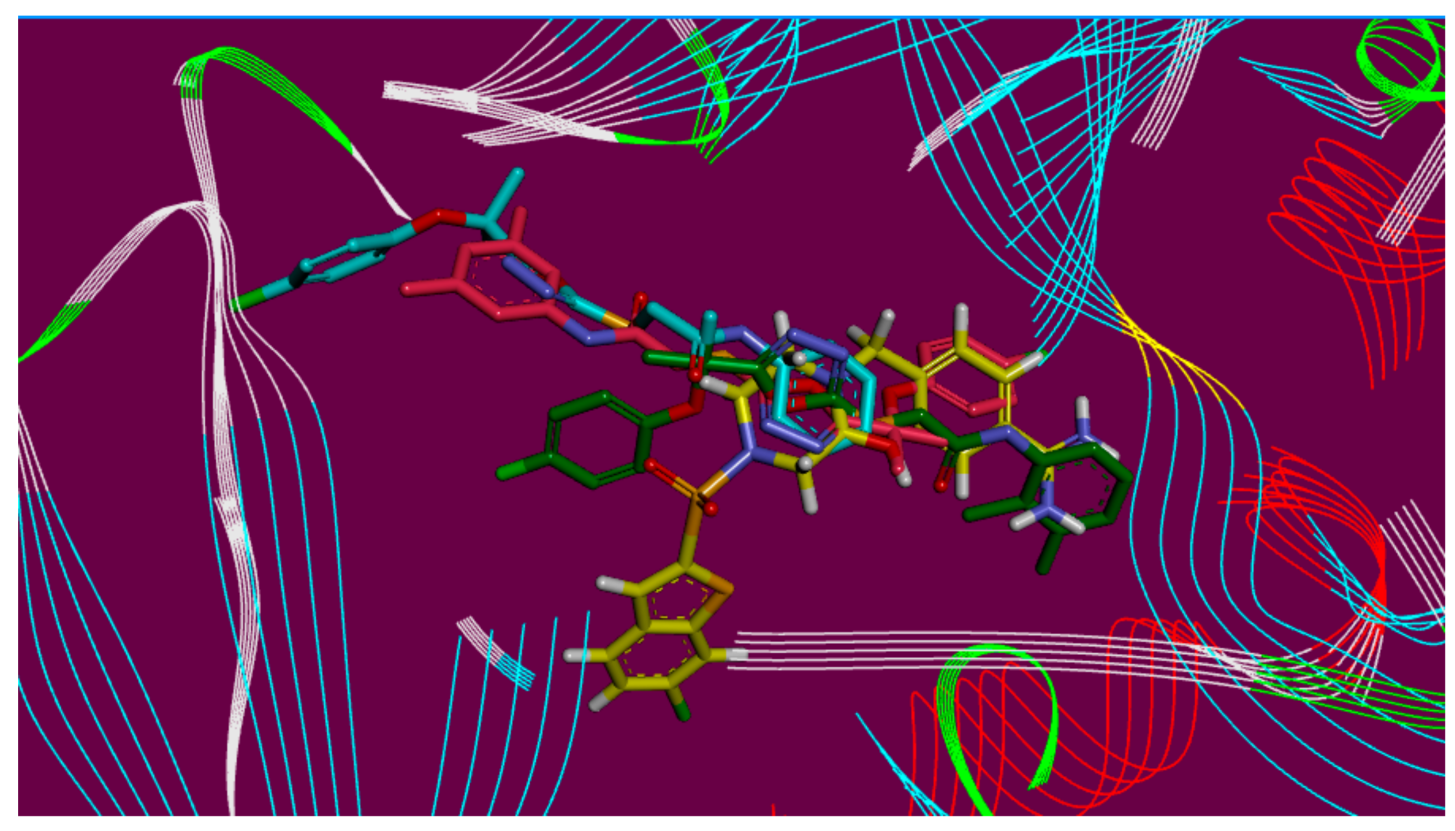
Overlap of bound conformations of RPR200095 (yellow) with compounds **3a** (blue), **3i** (pink), and **3e** (green).

**Figure 5:**
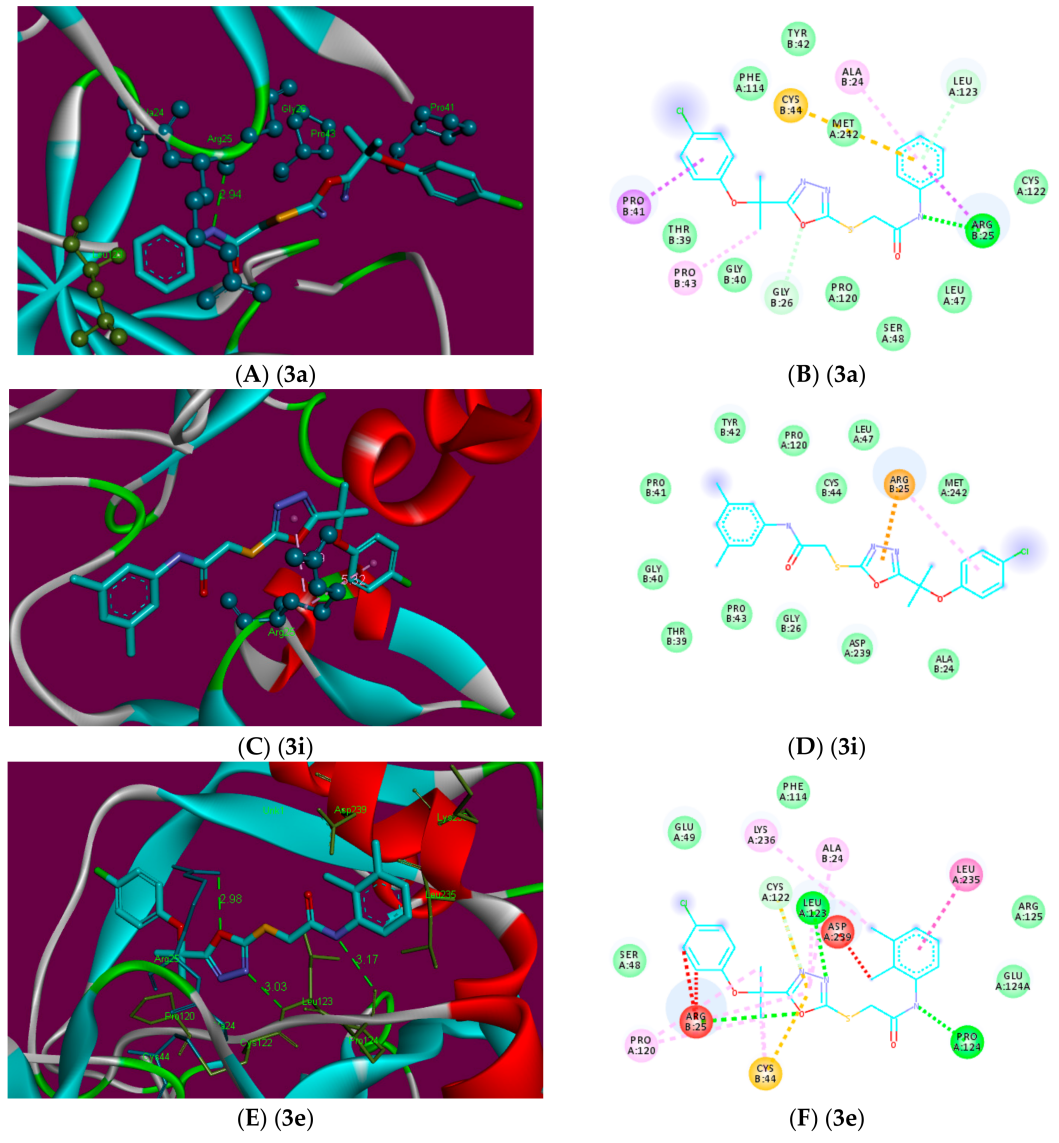
2D (**B**,**D**,**F**) and 3D (**A**,**C**,**E**) binding site interactions of the most probable docked ligands **3a**, **3i**, and **3e** within F-Xa active site showing their binding interactions with Patch Dock, depicting unfavorable bump (red), carbon hydrogen bond (light green), pi-alkyl (light pink), pi-cation (brown), and amide pi-stack (pink) interactions.

There are four binding pockets labeled as S1 to S4 within the active site. The most important are the S1 and S4 binding pockets that are exploited by Factor Xa inhibitors. The S1 pocket is in the form of a narrow cleft and it usually favors positively charged moieties such as benzamidine, amine, and guanidine [24]. S4 is the second main binding pocket shaped by different amino acid residues [25,26]. The energy score (S) was the main criterion to evaluate the binding affinity of ligand (Supplementary Table S1). The compound having highest binding affinity forms the most stable ligand-enzyme complex. The results of docking studies: energy score, involved factor Xa active site amino acid residues, and interacting ligands moieties for each compound and the reference inhibitor are given in Supplementary Table S1 and Figure 2, Figure 3, Figure 4 and Figure 5. Analysis of docking results showed that standard RPR200095 has a score of 5192 with an Atomic Contact Energy (ACE) value of −197.81 kcal/mol (Figure 2) when it docked with F-Xa and exhibited hydrophobic contact potential with pocket amino acids Lys^243^, Arg^25^, and Met^242^.

In the initial assessment of the docked complexes of F-Xa (1, RPR200095, **3a**–**3i**), five ligands **3a**, **3d**, **3e**, **3h**, and **3i** showed significant better interaction patterns when compared with RPR200095. These were found to bind near the entrance of active site gorge (Figure 2). However, compared to the binding of RPR200095, ligands **3a**, **3d**, **3e**, **3h**, and **3i** did not penetrate deeply into the binding pocket like RPR200095 instead, because of the bulkiness of these ligands, they fit on the top of the binding pocket. This might be blocking the substrate entry or releasing the products from active site showing antithrombotic activity.

Ligand **3a** showed the most potent interaction with the F-Xa active site with a score of 6270 and an ACE of −352.28 kcal/mol (Supplementary Table S1). Visual inspection of these complexes predicts a binding conformation of ligand **3a**, which showed significant interaction with the F-Xa binding site compared to the other ligands. The interacting residue of this complex is Arg^25^ (Figure 5). Ligand **3a** has shown a potential hydrogen bond between NH adjacent to carbonyl and phenyl groups and Arg^25^. The length of the hydrogen bond was 2.94 Å indicating significant interaction. Similarly, ligand **3a** exhibited hydrophobic contact potential with pocket amino acids Ala^24^, Pro^43^, and also depicted pi-sulfur contact potential with Cys^44^.

Interestingly, ligand **3i** showed no hydrogen bond interaction with the F-Xa receptor even given the significant geometric fit of this ligand in the receptor, and the scoring in Patch Dock is based on shape complementarily principles, it resulted in a score of 5612 and an ACE of −312.12 kcal/mol with Factor Xa (Figure 5). Ligand **3i** exhibited pi-cation contact potential with Arg^25^ and 1,3,4-oxadiazole, and a hydrophobic interaction was found between Arg^25^ and the 4-chlorophenyl-group. Ligand **3e** showed interaction with the F-Xa active site with a score of 5518 and an ACE of −189.68 kcal/mol. Ligand **3e** showed three hydrogen bonds between Arg^25^ and oxygen of 1,3,4-oxadiazole (2.98 Å), Leu^123^ and nitrogen of 1,3,4-oxadiazole (3.03 Å), Pro^124^ and the NH adjacent to the carbonyl and 2,3-dimethylphenyl groups (3.17 Å). Similarly, ligand **3e** exhibited hydrophobic contact potential with pocket amino acids Lys^236^, Ala^24^, Arg^25^, Leu^123^, and Cys^44^ respectively. Ligand **3e** has shown potential for pi-stack interaction with Leu^235^ and the 2,3-dimethylphenyl moiety (Figure 5).

Ligands **3d** and **3h** depicted the scores 5646 and 5612 with an ACE values −287.52 and −311.48 kcal/mol, respectively. This ligand **3d** showed a potential to accept a hydrogen bond from the backbone amino group Arg^25^ and sulfur attached with 1,3,4-oxadiazole (3.19 Å), Leu^123^, and nitrogen of 1,3,4-oxadiazole (3.25 Å) and also exhibited hydrophobic contact potential with pocket amino acids Cys^44^, Pro^120^, Arg^25^, Leu^235^, and Lys^236^ and can play an important part to give this ligand good binding affinity than other ligands of this series. Ligand **3h** has shown potential for van der Waals contact and such interactions involve the hydrophobic contact with Pro^43^, Leu^123^, and Phe^114^. This ligand depicted arene-cation contact with Arg^25^, also exhibited pi-sulfur contact with Met^242^, and 1,3,4-oxadiazoleand was unable to show polar interactions with the pocket amino acids.

In order to elaborate the structural elements liable for the observed inhibitory effect against the binding site F-Xa, the most active ligand **3a** bound to protein was assessed by Hyde utility of Lead IT software. Hyde allows visual approximation of favorable and unfavorable contributions due to the structure/bound conformation of inhibitor with the neighboring amino acids. The favorably contributing structural elements (atoms and torsions) to the overall binding energy colored blue, similarly the structural elements that are not contributing favorably are colored in red, and neutral elements are in white (Figure 3). The aromatic phenyl moieties substituted 1,3,4-oxadiazoles are contributing favorably to the binding energy. The only unfavorable structural element was the unsubstituted nitrogen atoms of the 1,3,4-oxadiazole ring. This led to the assumption that if these nitrogen atoms are substituted by some other atoms i.e., carbon or other hetero atoms, it may reason that it gains even better binding affinity and thereby show anticoagulant activity.

### 2.4. Computational Investigations

#### 2.4.1. Frontier Molecular Orbital (FMO) Analysis

**Figure 6:**
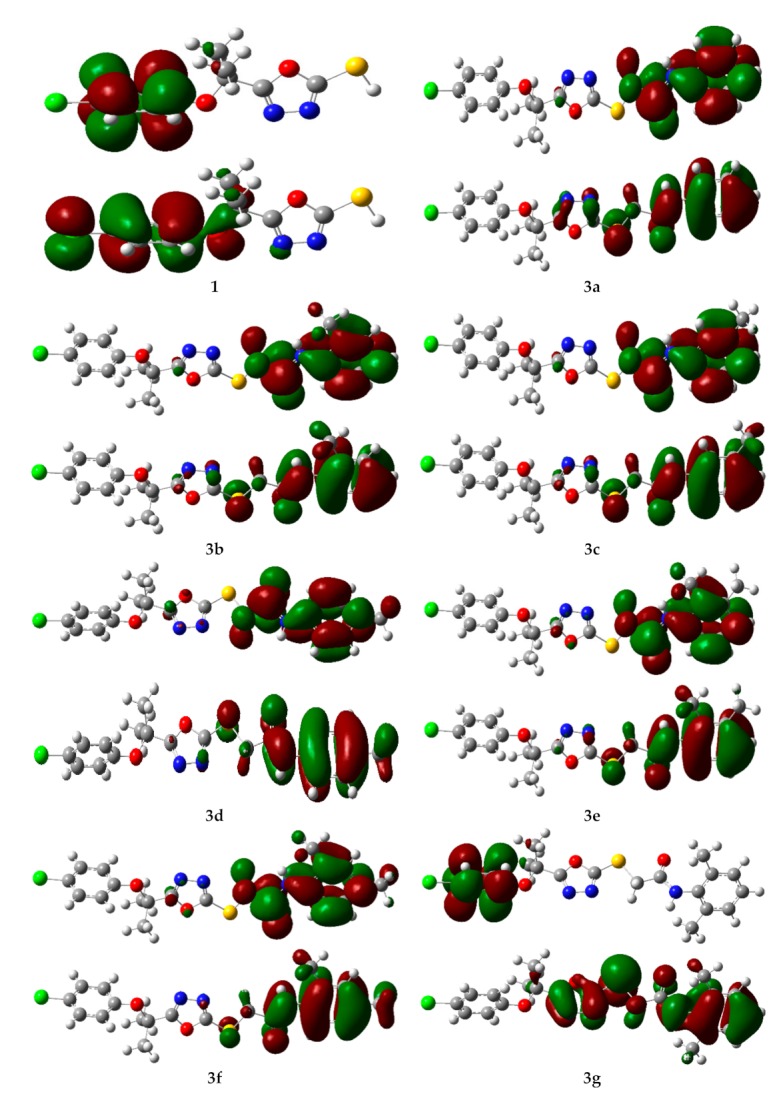

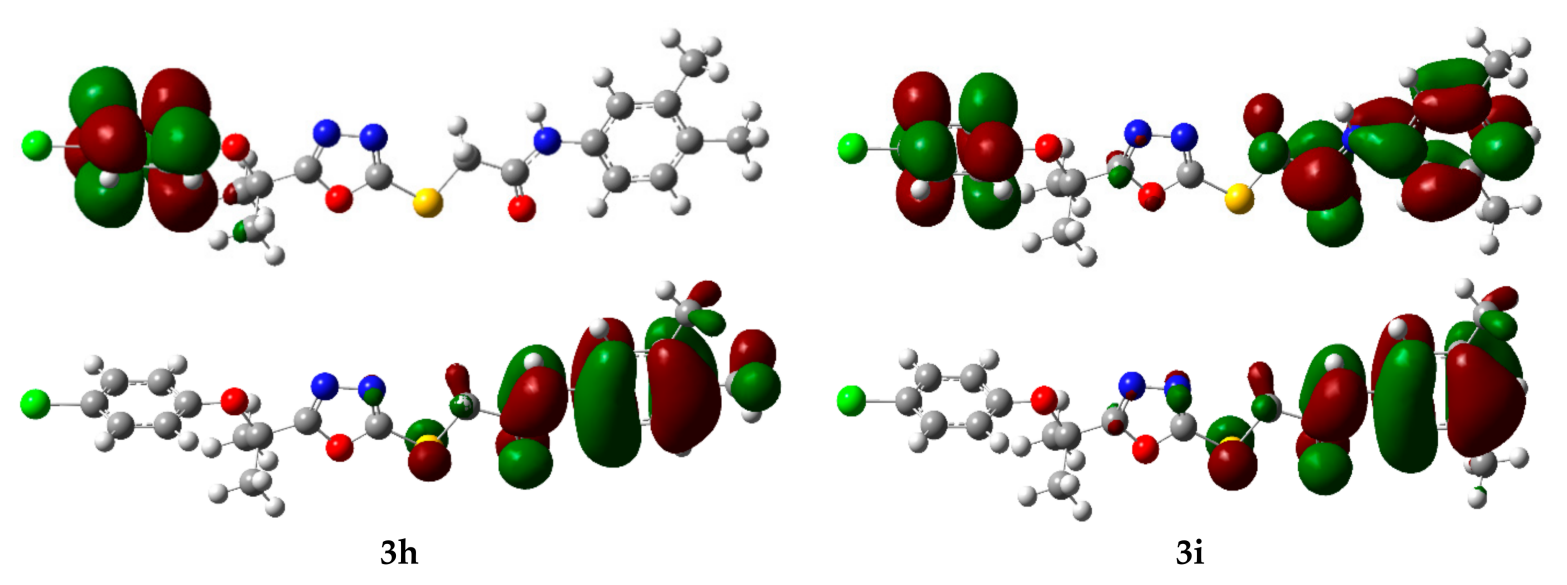
The charge density distribution of the HOMOs (bottom) and LUMOs (top) of the oxadiazoles derivatives (**3a**–**3i** and **1**). Color code of atoms: C—grey, Cl—light green, H—light grey, N—blue, O—red, S—yellow.

The energy of frontier orbital’s, namely, the Highest Occupied Molecular Orbital (HOMO) and Lowest Unoccupied Molecular Orbital (LUMO) are very popular parameters from quantum chemistry and calculations provide valuable information about molecular systems. The energy gap between HOMO and LUMO (*E_gap_*) measures the kinetic stability of the molecule [27]. A large value of the energy gap implies high kinetic stability and low chemical reactivity. Furthermore, the energy gap between HOMO and LUMO explains the intermolecular charge transfer (ICT) within the molecule, which is responsible for the bioactivity of the molecule. The distribution pattern of the FMOs has been illustrated in Figure 6. In all the studied derivatives **3a**–**3i**, except **1** in which HOMOs delocalized on 4-chlorophenoxygroup, the HOMOs had leading contribution from *N*-arylacetamide and the adjacent sulfur atom. Similarly, LUMOs also had a dominating contribution from the *N*-arylacetamide moiety except for **3g**, **3h**, and **1** which had a major contribution from the 4-chlorophenoxy group. However, in the case of **3i** HOMOs delocalized on both benzene rings along with acetamide groups. The ICT has been observed from *N*-arylacetamide to the 4-chlorophenoxy group units in **3g** and **3h**. 

The E_HOMO_, E_LUMO_, and HOMO-LUMO energy gaps (*E_gap_*) at the B3LYP/6-31G** level of theory has been tabulated in Supplementary Table S2. The highest *E_gap_* in **1** and **3g** corresponds to a decrease in their biological activity. While the low energy gap in **3a**, **3e**, **3d**, **3i**, and **3h** makes them potent inhibitors of F-Xa due and thus they show high anticoagulant activity.

Other electronic parameters such as ionization potential (I), electron affinity (A), hardness (η), softness (s), chemical potential (μ), absolute electronegativity (χ), electrophilicity index (ω), and dipole moment (D) of the synthesized compounds were also calculated, as seen in Supplementary Table S2. The electrophilicity index will be useful to explain the binding capacity with biomolecules [28].

#### 2.4.2. Molecular Electrostatic Potential (MEP)

**Figure 7:**
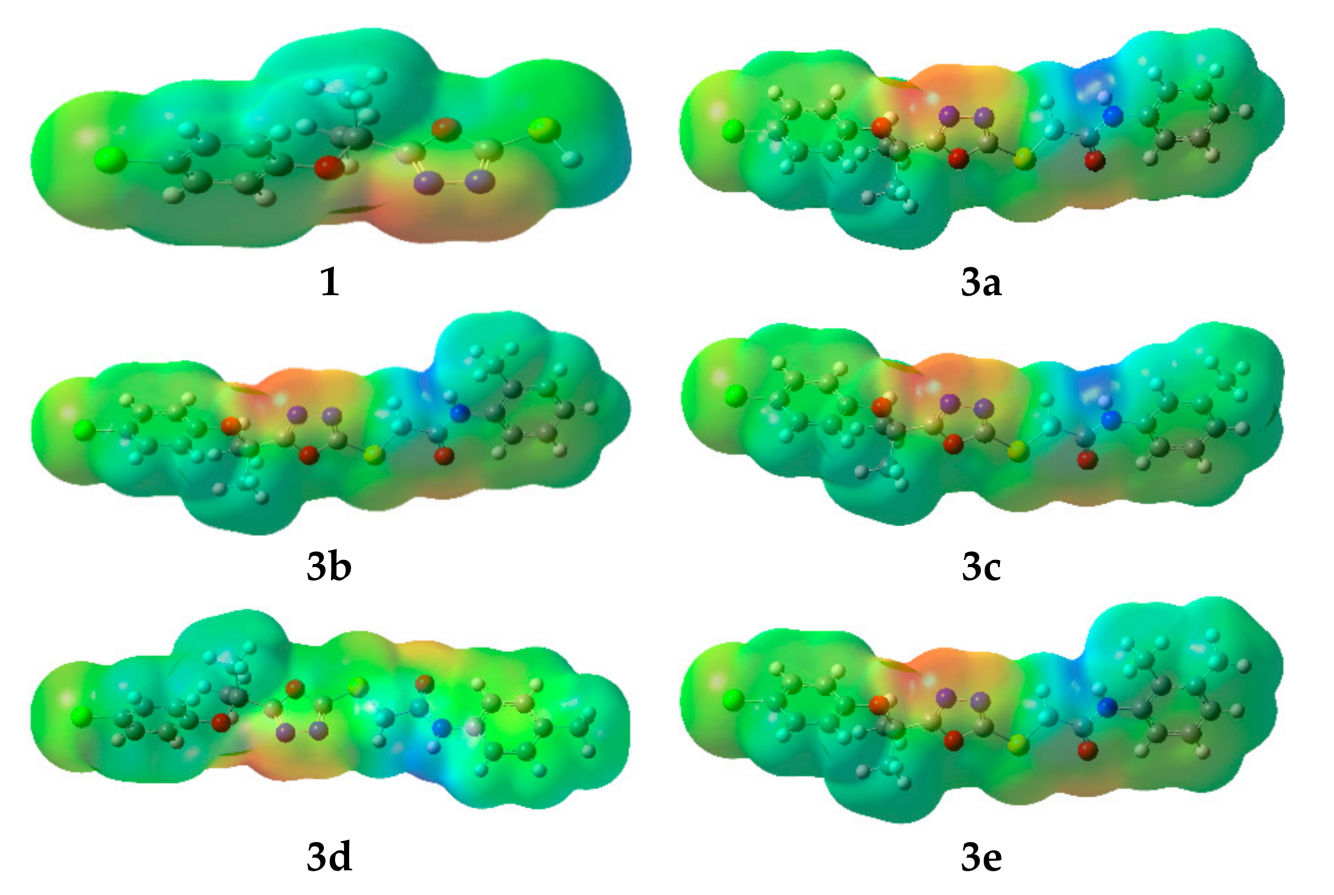

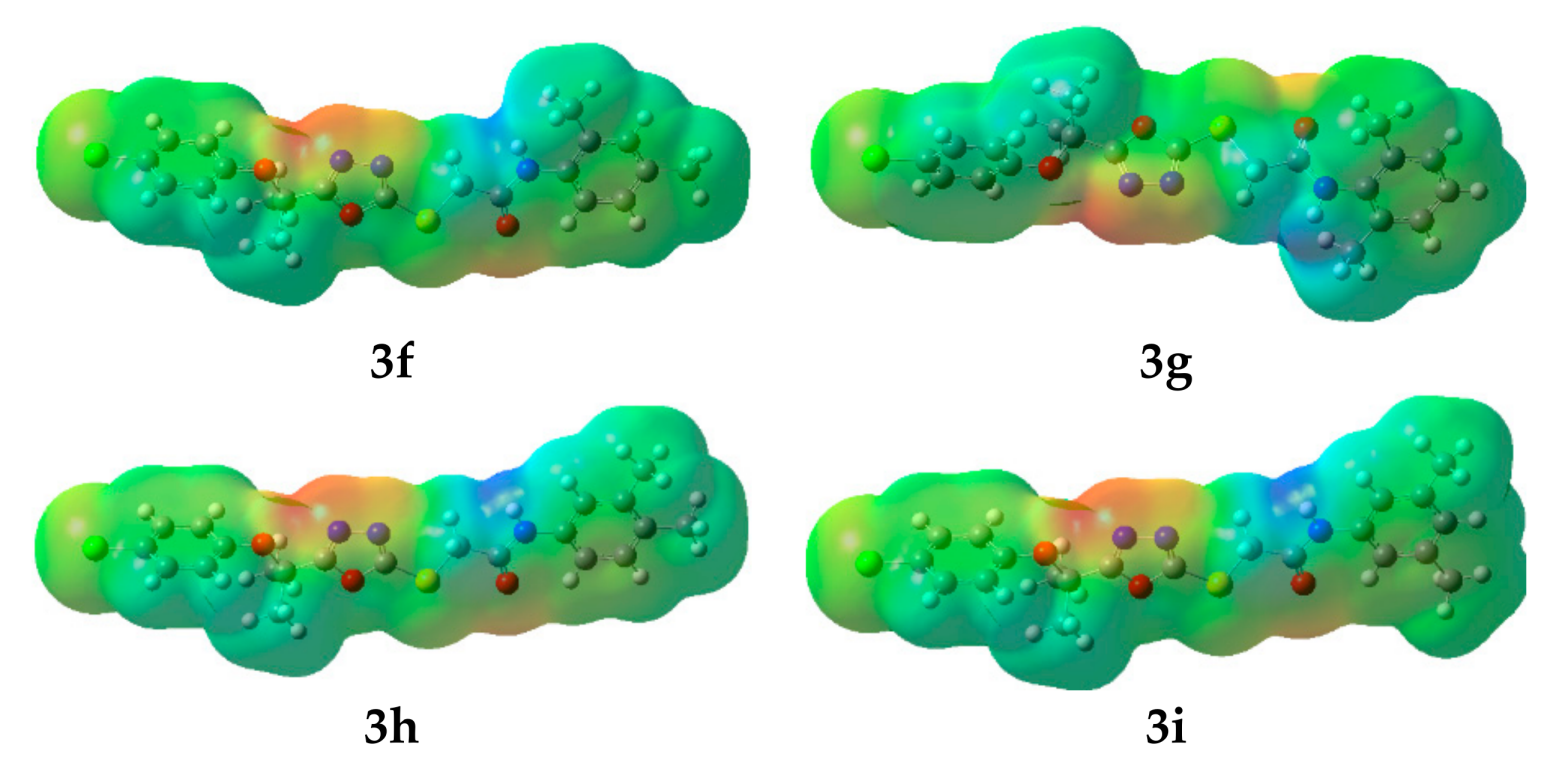
The molecular electrostatic potential surfaces of the oxadiazoles derivatives. Color code of atoms: C—grey, Cl—light green, H—light grey, N—blue, O—red, S—yellow.

Undoubtedly MEP is a very useful tool to understand molecular interactions. Especially, its 3-D mapping is widely used to explain the relative reactive sites for the electrophilic (negative region) and nucleophilic (positive region) attack in a molecule. It also provides visual understanding of the relative polarity of the molecule. To predict reactive sites for electrophilic and nucleophilic attack of all synthesized compounds, the MEP surface maps have been calculated and illustrated in Figure 7. The different colors represent the different values of the electrostatic potential at the surface. Negative and positive Electrostatic Potential (EP) regions are indicated by red and blue, respectively, while the neutral potential regions represented in green.

Careful analyses of the MEP revealed that the oxadiazole moiety would be a favorable site for electrophile attack in all the studied compounds. Further, in all synthesized derivatives the carboxamide group showed both positive and negative potential, making them good F-Xa inhibitors and anticoagulants.

## 3. Materials and Methods

### 3.1. Chemistry

Chemicals were purchased from Sigma Aldrich (St. Louis, MO, USA) and Alfa Aesar (Ward Hill, MA, USA). Melting points were taken on Griffin and George melting point apparatus using the open capillary tube method and were reported as uncorrected. Infrared spectra were recorded in KBr on a Jasco-320-A spectrophotometer. ^1^H-NMR signals were recorded on AVANCE AV-300 MHz, AVANCE AV-400 MHz, or AVANCE AV-500 MHz while ^13^C-NMR spectra were taken on a Bruker AVANCE AV-75 MHz, AVANCE AV-100 MHz and AVANCE AV-125 MHz spectrometer. EIMS signals were recorded on JEOL MS 600H-1 spectrometer. 5-[2-(4-Chlorophenoxy)propan-2-yl]-1,3,4-oxadiazol-2-thiol **1** [29] and aromatic *N*-substituted-2-bromoacetamides (**2a**–**2i**) were synthesized by previously reported methods with slight modifications [12].

#### 3.1.1. General Procedure for the Synthesis of *N*-Substituted 5-{[2-(4-Chlorophenoxy)propan-2-yl]-1,3,4-oxadiazol-2-ylthio}acetamides (**3a**–**3i**)

A mixture of 5-[2-(4-Chlorophenoxy)propan-2-yl]-1,3,4-oxadiazol-2-thiol **1** (0.271 g; 1 mmol) and lithium hydride (0.004 g; 2 mmol) was dissolved in DMF (10 mL) and the contents were subjected to ultrasound irradiations for 15 min followed by addition of *N*-substituted-2-bromoacetamide (1 mmol) with further irradiation till completion of the reaction as indicated by TLC (Ethylacetate:Hexanes, 1:4). Reaction times for different *N*-substituted 5-{[2-(4-chlorophenoxy)propan-2-yl]-1,3,4-oxadiazol-2-ylthio}acetamides varied from 1 to 1.5 h. After completion, the reaction mixture was poured on crushed ice. The precipitates were filtered, washed with distilled water, and dried to afford *N*-substituted 5-{[2-(4-chlorophenoxy)propan-2-yl]-1,3,4-oxadiazol-2-ylthio}acetamide. The products were recrystallized from 30% ethanol.

#### 3.1.2. 2-{5-[2-(4-Chlorophenoxy)propan-2-yl]-1,3,4-oxadiazol-2-ylthio}-*N*-phenylacetamide (**3a**)

The compound **3a** was obtained from the reaction of 5-[2-(4-chlorophenoxy)propan-2-yl]-1,3,4-oxadiazol-2-thiol **1** (0.271 g, 1 mmol) with 2-bromo *N*-phenylacetamide (0.213 g, 1 mmol) after 60 min. Shining white fluffy amorphous flakes; yield: 75% (0.302 g); m.p. 84–86 °C; IR (KBr, νmax, cm^−1^): 3138 (N–H str.), 3000 (C–H aromatic str.), 1671 (C=O amide str.), 1552 (C=C aromatic ring str.); ^1^H-NMR (500 MHz, DMSO-*d*_6_): δ (ppm) 10.42 (s, 1H, NH), 7.57 (d, *J* = 8 Hz, 2H, H-3′ & H-5′), 7.33 (t, *J* = 7.8 Hz, 2H, H-2″ & H-6″), 7.28 (d, *J* = 8.5 Hz, 2H, H-3″ & H-5″), 7.08 (t, *J* = 7.3 Hz, 1H, H-4″), 6.75 (d, *J* = 9 Hz, 2H, H-2′ & 6′), 4.33 (s, 2H, S-***CH***_2_-CO), 1.71 (s, 6H, C(***CH***_3_)_2_) (Figure S1); ^13^C-NMR (125 MHz, DMSO-*d*_6_, δ/ppm): 168.85 (C=O), 165.17 (C-2), 164.90 (C-5), 153.29 (C-1′), 139.10 (C-1″), 129.93 (C-4′), 129.76 (C-3′ & C-5′), 129.32 (C-3″ & C-5″), 128.56 (C-4″), 124.18 (C-2″ & C-6″), 119.62 (C-2′ & C-6′), 75.99 (***C***(***CH***_3_)_2_), 37.32 (S-***CH***_2_-CO), 25.70 (C(***CH***_3_)_2_) (Figure S2); EIMS: *m*/*z* 403 [M^+^], 405 [M^+^ + 2] (Figure S3).

#### 3.1.3. 2-{5-[2-(4-Chlorophenoxy)propan-2-yl]-1,3,4-oxadiazol-2-ylthio}-*N*-(2-methylphenyl)acetamide (**3b**)

The compound **3b** was synthesized by the reaction of 5-[2-(4-chlorophenoxy)propan-2-yl]-1,3,4-oxadiazol-2-thiol **1** (0.271 g, 1 mmol) with 2-bromo-*N*-(2-methylphenyl)acetamide (0.227 g, 1 mmol) after a period of 1 h and 20 min. Light yellow color powder; yield: 87% (0.362 g); m.p. 140–142 °C; IR (KBr, νmax, cm^−1^): 3272 (N–H, str.), 2965 (C–H, str. of aromatic ring), 1644 (C=O amide str.), 1383 (C=C, aromatic str.); ^1^H-NMR (500 MHz, DMSO-*d*_6_): δ (ppm) 10.48 (s, 1H, NH), 7.38–7.32 (m, 5H, H-3′, H-5′, H-4″, H-5″ & H-6″), 7.22 (d, *J* = 8 Hz, 1H, H-3″), 6.94 (d, *J* = 9 Hz, 2H, H-2′ & H-6′), 4.26 (d, *J* = 17.5 Hz, 1H, *Ha*), 4.19 (d, *J* = 17.5 Hz, 1H, *Hb*), 2.18 (s, 3H, Ar-CH_3_), 1.47, 1.46 (s, C(***CH***_3_)_2_) (Figure S4); ^13^C-NMR (125 MHz, DMSO-*d*_6_, δ/ppm): 171.80 (C=O), 169.58 (C-2), 164.64 (C-5), 154.10 (C-1′), 136.48 (C-1″), 134.57 (C-2″), 131.26 (C-3″), 129.71 (C-5″), 129.46 (C-3′ & C-5′), 129.15 (C-4′), 127.31 (C-4″), 126.40 (C-6″), 121.91 (C-2′ & C-6′), 80.83 (***C***(CH_3_)_2_), 33.08 (S-***CH_2_***-CO), 25.47, 25.44 (C(***CH***_3_)_2_), 17.56 (Ar-CH_3_) (Figure S5); EIMS: *m*/*z* 417 [M^+^], 419 [M^+^ + 2] (Figure S6).

#### 3.1.4. 2-{5-[2-(4-Chlorophenoxy)propan-2-yl]-1,3,4-oxadiazol-2-ylthio}-*N*-(3-methylphenyl)acetamide (**3c**)

The compound **3c** was achieved from the reaction of 5-[2-(4-chlorophenoxy) propan-2-yl]-1,3,4-oxadiazol-2-thiol **1** (0.271 g, 1 mmol) with 2-bromo-*N*-(3-methylphenyl)acetamide (0.227 g, 1 mmol) after a period of 1 h and 15 min. Lemon yellow color powder; yield: 78% (0.325 g); m.p. 71–72 °C; IR (KBr, νmax, cm^−1^): 3155 (N–H, str.), 2989 (C–H, str. of aromatic ring), 1655 (C=O amide str.), 1488 (C=C, aromatic str.); ^1^H-NMR (500 MHz, CDCl_3_): δ (ppm) 8.93 (s, 1H, NH), 7.35 (s, 1H, H-2″), 7.27 (d, *J* = 8.5 Hz, 1H, 6″), 7.19 (t, *J* = 7.8 Hz, 1H, 5″), 7.11–7.10 (m, 2H, H-3′ & H-5′), 6.93 (d, *J* = 7.5 Hz, 1H, H-4″), 6.65–6.63 (m, 2H, H-2′ & H-6′), 3.97 (s. 2H, S-***CH***_2_-CO), 2.32 (s, 3H, Ar-CH_3_), 1.77 (s, 6H, C(***CH***_3_)_2_) (Figure S7); ^13^C-NMR (100 MHz, DMSO-*d*_6_, δ/ppm): 169.88 (C=O), 166.17 (C-2), 165.02 (C-5), 152.84 (C-1′), 139.04 (C-3″), 137.43 (C-1″), 129.41 (C-3′ & C-5′), 128.87 (C-5″), 125.61 (C-4′), 122.98 (C-2′, C-6′ & C-6″), 120.46 (C-4″), 116.99 (C-2″), 75.55 (***C***(***CH***_3_)_2_), 36.23 (S-***CH***_2_-CO), 25.86 (C(***CH***_3_)_2_), 21.44 (Ar-CH_3_) (Figure S8); EIMS: *m*/*z* 417 [M^+^], 419 [M^+^ + 2] (Figure S9).

#### 3.1.5. 2-{5-[2-(4-Chlorophenoxy)propan-2-yl]-1,3,4-oxadiazol-2-ylthio}-*N*-(4-methylphenyl)acetamide (**3d**)

The compound **3d** was obtained from the reaction of 5-[2-(4-chlorophenoxy)propan-2-yl]-1,3,4-oxadiazol-2-thiol **1** (0.271 g, 1 mmol) with 2-bromo-*N*-(4-methylphenyl)acetamide (0.227 g, 1 mmol) after 60 min. Dirty white color powder; yield: 84% (0.350 g); m.p. 68–70 °C; IR (KBr, νmax, cm^−1^): 3313 (N–H, stretching), 2979 (C–H, str. of aromatic ring), 1676 (C=O amide str.), 1483 (C=C, aromatic str.); ^1^H-NMR (500 MHz, DMSO-*d*_6_): δ (ppm) 10.33 (s, 1H, NH), 7.46 (d, *J* = 8 Hz, 2H, H-3′ & H-5), 7.27 (d, *J* = 9 Hz, 2H, H-2′ & H-6′), 7.12 (d, *J* = 8 Hz, 2H, H-2″ & H-6″), 6.75 (d, *J* = 9 Hz, 2H, H-3″ & H-5″), 4.31 (s, 2H, S-***CH***_2_-CO), 2.25 (s, 3H, Ar-CH**_3_**), 1.71 (s, 6H, C(***CH***_3_)_2_) (Figure S10); ^13^C-NMR (125 MHz, DMSO-*d*_6_, δ/ppm): 168.84 (C=O), 164.91 (C-2), 164.90 (C-5), 153.29 (C-1′), 136.60 (C-4″), 133.13 (C-1″), 129.75 (C-3′ & C-5′), 129.68 (C-3″ & C-5″), 128.57 (C-4′), 124.18 (C-2″ & C-6″), 119.64 (C-2′ & C-6′), 75.98 (***C***(***CH***_3_)_2_), 37.31 (S-***CH***_2_-CO), 25.70 (C(***CH***_3_)_2_), 20.91 (Ar-CH_3_) (Figure S11); EIMS: *m*/*z* 417 [M^+^], 419 [M^+^ + 2] (Figure S12).

#### 3.1.6. 2-{5-[2-(4-Chlorophenoxy)propan-2-yl]-1,3,4-oxadiazol-2-ylthio}-*N*-(2,3-dimethylphenyl)acetamide (**3e**)

The compound **3e** was obtained from the reaction of 5-[2-(4-chlorophenoxy)propan-2-yl]-1,3,4-oxadiazol-2-thiol **1** (0.271 g, 1 mmol) with 2-bromo-*N*-(2,3-dimethylphenyl)acetamide (0.241 g, 1 mmol) after 1 h and 30 min. Creamy white color powder; yield: 86% (0.371 g); m.p. 186–188 °C; IR (KBr, νmax, cm^−1^): 3300 (N–H, stretching), 2952 (C–H, str. of aromatic ring), 1645 (C=O amide str.), 1392 (C=C, aromatic str.); ^1^H-NMR (500 MHz, DMSO-*d*_6_): δ (ppm) 10.45 (s, 1H, NH), 7.34 (d, *J* = 9 Hz, 2H, H-3′ & H-5′), 7.27 (d, *J* = 7.5 Hz, 1H, H-6″), 7.21 (t, *J* = 7.6 Hz, 1H, H-5″), 7.04 (d, *J* = 8 Hz, 1H, H-4″), 6.94 (d, *J* = 9 Hz, 2H, H-2′ & H-6′), 4.26 (d, *J* = 17.5 Hz, 1H, *Ha*), 4.18 (d, *J* = 7 Hz, 1H, *Hb*), 2.31 (s, 3H, Ar-CH_3_), 2.03 (s, 3H, Ar-CH_3_), 1.47, 1.46 (s, 6H, C(***CH***_3_)_2_) (Figure S13); ^13^C-NMR (125 MHz, DMSO-*d*_6_, δ/ppm): 171.87 (C=O), 169.49 (C-2), 164.72 (C-5), 154.07 (C-1′), 138.23 (C-6″), 134.94 (C-1″), 134.58 (C-3″), 130.93 (C-4′), 129.45 (C-3′ & C-5′), 126.68 (C-2″), 126.60 (C-4″), 126.38 (C-5″), 121.90 (C-2′ & C-6′), 80.82 (***C***(***CH***_3_)_2_), 33.03 (S-***CH***_2_-CO), 25.45 (C(***CH***_3_)_2_), 20.36 (Ar-CH_3_), 14.22 (Ar-CH_3_) (Figure S14); EIMS: *m*/*z* 431 [M^+^], 433 [M^+^ + 2] (Figure S15).

#### 3.1.7. 2-{5-[2-(4-Chlorophenoxy)propan-2-yl]-1,3,4-oxadiazol-2ylthio}-*N*-(2,4-dimethylphenyl)acetamide (**3f**)

The compound **3f** was obtained from the reaction of 5-[2-(4-chlorophenoxy)propan-2-yl]-1,3,4-oxadiazol-2-thiol **1** (0.271 g, 1 mmol) with 2-bromo-*N*-(2,4-dimethylphenyl)acetamide (0.241 g, 1 mmol) after 1 h and 20 min. Golden yellow color powder; yield: 81% (0.349 g); m.p. 78–80 °C; IR (KBr, νmax, cm^−1^): 3260 (N–H, stretching), 2980 (C–H, str. of aromatic ring), 1632 (C=O amide str.), 1381 (C=C, aromatic str.); ^1^H-NMR (CDCl_3_, 500 MHz): δ (ppm) 8.59 (s, 1H, NH), 7.69 (d, *J* = 8 Hz, 1H, H-6″), 7.11–7.09 (m, 2H, H-3′ & H-5′), 6.98–6.97 (m, 2H, H-3″ & H-5″), 6.65–6.63 (m, 2H, H-2′ & H-6′), 4.01 (s, 2H, S-***CH***_2_-CO), 2.27 (s, 3H, Ar-CH_3_), 2.16 (s, 3H, Ar-CH_3_), 1.77 (s, 6H, C(***CH***_3_)_2_) (Figure S16); ^13^C-NMR (125 MHz, CDCl_3_, δ/ppm): 169.93 (C=O), 166.13 (C-2), 165.32 (C-5), 152.87 (C-1′), 135.19 (C-4″), 132.85 (C-2″), 131.24 (C-1″), 129.39 (C-3′ & C-5′), 129.17 (C-3″), 127.23 (C-5″), 122.83 (C-2′, C-6′ & C-6″), 122.66 (C-4′), 75.51 (***C***(CH_3_)_2_), 35.97 (S-***CH***_2_-CO), 25.88 (C(***CH***_3_)_2_), 20.85 (Ar-CH_3_), 17.85 (Ar-CH_3_) (Figure S17); EIMS: *m*/*z* 431 [M^+^], 433 [M^+^ + 2] (Figure S18).

#### 3.1.8. 2-{5-[2-(4-Chlorophenoxy)propan-2-yl]-1,3,4-oxadiazol-2-ylthio}-*N*-(2,6-dimethylphenyl)acetamide (**3g**)

The compound **3g** was obtained from the reaction of 5-[2-(4-chlorophenoxy)propan-2-yl]-1,3,4-oxadiazol-2-thiol **1** (0.271 g, 1 mmol) with 2-bromo-*N*-(2,6-dimethylphenyl)acetamide (0.241 g, 1 mmol) after 1 h. Creamy white color powder; yield: 89% (0.383 g); m.p. 98–100 °C; IR (KBr, νmax, cm^−1^): 3330 (N–H, stretching), 2958 (C–H, str. of aromatic ring), 1661 (C=O amide str.), 1482 (C=C, aromatic str.); ^1^H-NMR (500 MHz, DMSO-*d*_6_): δ (ppm) 9.71 (s, 1H, NH), 7.26 (d, *J* = 9 Hz, 2H, H-3′ & H-5′), 7.09–7.06 (m, 3H, H-3″, H-4″ & H-5″), 6.76 (d, *J* = 9 Hz, 2H, H-2′ & H-6′), 4.34 (s, 2H, S-***CH***_2_-CO), 2.13 (s, 6H, Ar-2CH_3_), 1.73 (s, 6H, C(***CH***_3_)_2_) (Figure S19); ^13^C-NMR (125 MHz, DMSO-*d*_6_, δ/ppm): 168.9 (C=O), 164.98 (C-2), 164.94 (C-5), 153.31 (C-1′), 135.60 (C-2″ & C-6″), 135.01 (C-1″), 129.76 (C-3′ & C-5′), 128.54 (C-4″), 128.18 (C-3″ & C-5″), 127.14 (C-4′), 124.15 (C-2′ & C-6′), 75.98 (***C***(CH_3_)_2_), 36.25 (S-***CH***_2_-CO), 25.75 (C(***CH***_3_)_2_), 18.47 (Ar-2CH_3_) (Figure S20); EIMS: *m*/*z* 431 [M^+^], 433 [M^+^ + 2] (Figure S21).

#### 3.1.9. 2-{5-[2-(4-Chlorophenoxy)propan-2-yl]-1,3,4-oxadiazol-2-ylthio}-*N*-(3,4-dimethylphenyl)acetamide (**3h**)

The compound **3h** was obtained from the reaction of 5-[2-(4-chlorophenoxy)propan-2-yl]-1,3,4-oxadiazol-2-thiol **1** (0.271 g, 1 mmol) with 2-bromo-*N*-(3,4-dimethylphenyl)acetamide (0.241 g, 1 mmol) after 1 h and 10 min. Off-white color powder; yield: 91% (0.392 g); m.p. 80–82 °C; IR (KBr, νmax, cm^−1^): 3357 (N–H, stretching), 2904 (C–H, str. of aromatic ring), 1640 (C=O amide str.), 1380 (C=C, aromatic str.); ^1^H-NMR (300 MHz, DMSO-*d*_6_): δ (ppm) 10.22 (s, 1H, NH), 7.33 (s, 1H, H-2″), 7.26–7.22 (m, 2H, H-5″ & H-6″), 7.04 (d, *J* = 8 Hz, 2H, H-3′ & H-5′), 6.75–6.71 (m, 2H, H-2′, & H-6′), 4.27 (s, 2H, S-***CH***_2_-CO), 2.16 (d, *J* = 4.5 Hz, 6H, Ar-2CH_3_), 1.69 (s, 6H, C(***CH***_3_)_2_) (Figure S22); ^13^C-NMR (75 MHz, DMSO-*d*_6_, δ/ppm): 168.37 (C=O), 164.38 (C-2), 164.35 (C-5), 152.79 (C-1′), 136.42 (C-3″), 136.32 (C-1″), 131.47 (C-4″), 129.63 (C-5″), 129.24 (C-3′ & C-5′), 128.08 (C-4′), 123.74 (C-2′ & C-6′), 120.39 (C-2″), 116.72 (C-6″), 75.51 (***C***(CH_3_)_2_), 36.79 (S-***CH***_2_-CO), 25.22 (C(***CH***_3_)_2_), 19.75 (Ar-CH_3_), 18.75 (Ar-CH_3_) (Figure S23); EIMS: *m/z* 431 [M^+^], 433 [M^+^ + 2] (Figure S24).

#### 3.1.10. 2-{5-[2-(4-Chlorophenoxy)propan-2-yl]-1,3,4-oxadiazol-2-ylthio}-*N*-(3,5-dimethylphenyl)acetamide (**3i**)

The compound **3i** was obtained from the reaction of 5-[2-(4-chlorophenoxy)propan-2-yl]-1,3,4-oxadiazol-2-thiol **1** (0.271 g, 1 mmol) with 2-bromo-*N*-(3,5-dimethylphenyl)acetamide (0.241 g, 1 mmol) after 1 h and 30 min. Off-white color powder; yield: 85% (0.366 g); m.p. 122–124 °C; IR (KBr, νmax, cm^−1^): 3299 (N–H, stretching), 2995 (C–H, str. of aromatic ring), 1680 (C=O amide str.), 1580 (C=C, aromatic str.); ^1^H-NMR (500 MHz, DMSO-*d*_6_): δ (ppm) 10.27 (s, 1H, NH), 7.27 (d, *J* = 8.5 Hz, 2H, H-3′ & H-5′), 7.20 (s, 2H, H-2″ & H-6″), 6.75 (d, *J* = 8.5 Hz, 2H, H-2′ & H-6′), 6.72 (s, 1H, H-4″), 4.29 (s, 2H, S-***CH***_2_-CO), 2.23 (s, 6H, Ar-2CH_3_), 1.70 (s, 6H, C(***CH***_3_)_2_) (Figure S25); ^13^C-NMR (125 MHz, DMSO-*d*_6_, δ/ppm): 168.3 (C=O), 165.03 (C-2), 164.90 (C-5), 153.27 (C-1′), 138.96 (C-1″), 138.29 (C-3″ & C-5″), 129.73 (C-3′ & C-5′), 128.59 (C-4″), 125.69 (C-4′), 124.27 (C-2″ & C-6″), 11.39 (C-2′ & C-6′), 76.00 (***C***(CH_3_)_2_), 37.31 (S-***CH***_2_-CO), 25.68 (C(***CH***_3_)_2_), 21.53 (Ar-2CH**_3_**) (Figure S26); EIMS: *m*/*z* 431 [M^+^], 433 [M^+^ + 2] (Figure S27).

### 3.2. Biological Assay

#### 3.2.1. Sample Solution Preparation

A 100 mg each of each synthetic compound was mixed in 10 mL distilled water and shaken vigorously then stirred overnight. The resulting solution was then filtered by using 0.22 μm syringe filter. One-hundred micro liters of this aqueous preparation was added to the pre-weighed Eppendorf tube containing the clots to check thrombolytic activity.

#### 3.2.2. In Vitro Antithrombotic Activity

In vitro antithrombotic activity was carried as reported earlier [30]. Briefly, venous blood was drawn from the healthy volunteers both male and female (20–30 years) with no history of anticoagulant therapy or oral contraceptive and no smoking history. Half a milliliter was used in pre-weighed sterile Eppendorf tubes and incubated at 37 °C for 45 min. After clot formation, serum was completely removed without disturbing the clot and weighed again to determine the clot weight. One-hundred microliters of each of the tested compounds was added separately. Streptokinase (SK) 1,500,000 IU (Square Pharmaceuticals Ltd., Dhak, Bangladesh) used as positive control (100 µL) while sterile distilled water (100 µL) was used as negative control. All the tubes were then incubated at 37 °C for 90 min. After incubation, fluid released was removed carefully and weighed to observe the difference in weight after clot disruption. The difference obtained in weight taken before and after clot lysis was expressed as percentage of clot lysis [31]. The experiment was repeated three times with the blood samples of the 12 volunteers with their signed consent and the experiment was approved by Institutional Ethical Committee (Approval No. D/025/2018, 7 March 2018). The following formula was used to determine the percentage of clot lysis.

Percentage of clot lysis (%) = [Initial clot weight-Final clot weight/Initial weight of clot] × 100

#### 3.2.3. In Vivo Antithrombotic Activity 

##### Experimental Animals

Sprague Dawley (SD) rats (100–120 g) were used to determine the in vivo anticoagulation effects of tested compounds and kept under control temperature (25 ± 5 °C) and humidity (50 ± 10%) in an animal house, with free access of pathogen and autoclave tap water for 24 h. Experiments were approved by Institutional Ethical Committee, University of the Punjab, Lahore (Approval No. D/025/2018, 7 March 2018) and international ethical guidelines were also followed for the care of laboratory animals to provide them with a healthy environment.

##### In Vivo Clotting Time Determination

Blood CT determination was used to evaluate the in vivo antithrombotic activity of synthetic compounds. Previously established method with slight modifications was used to estimate the CT [7,8]. SD Rats were divided into twelve groups and each group contained six rats. The first group was given 0.5% carboxy methyl cellulose (CMC) orally and served as the negative control. Rats in the second groups were designated as positive controls and received 500 IU/kg unfractionated heparin orally. The rats in other groups were given the tested compounds (**3a**–**3i**) and suspended in 0.5% CMC orally (25 mg/kg). Blood was drawn from the tail of each rat to check the CT. The time course effect was determined CT and measured at regular intervals of one hour.

### 3.3. Molecular Docking Studies

In silico analysis of the newly designed 1,3,4-oxadiazoles derivatives against F-Xa protein were carried out. The crystal structure of the F-Xa protein was retrieved from the Research Collaboratory for Structural Bioinformatics (RCSB) Protein Data Bank (PDB ID 1NFY). The experimental (in vivo) studies were carried out against Factor Xa (Ec: 3.4.21.6) from Sprague Dawley rats and the docking study against human F-Xa (PDB ID: 1NFY at 2.1 Å resolution).

#### 3.3.1. Preparation of Target F-Xa and Compounds for Docking

The coordinate files were subjected to Discovery Studio 4.5 Visualizer for pre-docking receptor preparation by removing water molecules and adding hydrogen atoms. Ligands **1**, RPR200095, and **3a**–**3i** were docked with F-Xa (1NFY) by Patch Dock (http://bioinfo3d.cs.tau.ac.il/PatchDock/) to find the docking transformations that produced a good molecular shape complementarily based on shape complementarily principles [32]. The input files included the receptor protein and ligand in PDB format.

Patch Dock offers multiple solutions and “solution 1” was selected as it surrounded the most crucial residues for the binding pocket for docking analyses assigned in crystal structure of F-Xa target site (1NFY) [23]. The docked structures were examined by using Discovery Studio 4.5 Visualizer and Chimera 1.9.

#### 3.3.2. Docking Analysis

The binding affinities of the docked ligands were evaluated as scores and Atomic Contact Energy (ACE) of the docked complexes. The hydrogen bonding and hydrophobic interactions of each ligand were assessed within the binding pocket of the receptor protein. The conformation of the ligands with highest biological activities is showed in Supplementary Table S2 and Figure 2, Figure 3, Figure 4 and Figure 5 with their favorable contacts in the binding pockets. To get qualitative evaluation and to recognize molecular basis of the calculated biological activities, the docked complexes of ligands **1**, RPR200095, and **3a**–**3i** were investigated.

### 3.4. Computational Methodology

In this study, all the computational calculations (including representation of highest HOMO and LUMO in the checkpoint files) were performed by Gaussian 09 software [33] with the Becke’s three parameter hybrid exchange functional [34] and Lee–Yange–Parr correlation functionals (B3LYP) [35,36]. The geometry of all the structures were optimized using B3LYP/6-31G** basis set. To ensure that the optimized geometry actually corresponds to the equilibrium (minimum energy) structure, the harmonic vibrational frequency analysis was also performed at same basic set level to detect any imaginary frequency. The Gauss view software package was used to visualize the computed structures including HOMO, LUMO, and molecular electrostatic potential (MEP) representations.

## 4. Conclusions

The carboxamide group is present in many F-Xa inhibitor agents; a set of novel compounds 5-[2-(4-chlorophenoxy)propan-2-yl]-1,3,4-oxadiazol-2-thiol derivatives (**3a**–**3i**) were synthesized and characterized to confirm their structures, and were determined to be ameliorative with regards to in vitro, in vivo, and in silico efficacy against thrombosis. These derivatives in addition to the main scaffold 1,3,4-oxadiazole contained carboxamide entity which might be involved to suppress the thrombosis formation in CVDs and CHD. Inhibition of F-Xa factor by oxadiazole derivatives to ameliorate the blood coagulation is one the major pharmaceutical approach [18,19]. The newly synthesized 1,3,4-oxadiazole derivatives depicted impressive in vitro antithrombotic effects compared with Oxadiazole (**1**). While in vivo results taken from rats by CT values determination showed impassive antithrombotic potential especially compound **3a**. The in vitro, in vivo and in silico results were supported by previously reported research where coumarinyl oxadiazole derivatives suppressed thrombosis [17,23,31]. Docking results revealed that ligands (**3a**, **3e**, **3i**, **3d** and **3h**) have potent inhibitory potential against F-Xa and higher docking scores than the control ligand RPR200095. In silico results are good in agreement with the anti-thrombotic activity of the tested compounds (**3a**, **3e**, **3i**, **3d** and **3h**) that were far superior than standard drug unfractionated heparin. Results strongly suggest that these compounds have potent anti-thrombotic potential in preventing arterial thrombosis and might be used as effective anticoagulant agent. The powerful antithrombotic action of 5-[2-(4-chlorophenoxy)propan-2-yl]-1,3,4-oxadiazol-2-thiol derivatives calls for further comprehensive pharmacological research into their long term toxic effects and their protective effects.

## Figures and Tables

**Table 1:** Different substitutions in **3a**–**3i**.

Codes	3a	3b	3c	3d	3e	3f	3g	3h	3i
R^1^	H	H	H	H	2-CH_3_	2-CH_3_	2-CH_3_	3-CH_3_	3-CH_3_
R^2^	H	2-CH_3_	3-CH_3_	4-CH_3_	3-CH_3_	4-CH_3_	6-CH_3_	4-CH_3_	5-CH_3_

**Table 2:** In vitro clot lysis effect of synthetic compounds on human blood.

Sr. No.	Compounds	Clotlysis (%)
1	**1**	20.2
2	**3a**	27
3	**3b**	20.5
4	**3c**	11
5	**3d**	25
6	**3e**	32
7	**3f**	25
8	**3g**	20.6
9	**3h**	21
10	**3i**	41
11	Distilled water	4
12	Streptokinase (SK)	38

## Supplementary Materials

^1^H-NMR spectra, ^13^C-NMR spectra, EI-MS of compounds **3a**–**3i**, Tables S1 and S2 are provided. Supplementary materials can be found at http://www.mdpi.com/1422-0067/19/11/3606/s1.
