# Supplementary material for: Molecular Docking, Computational, and Antithrombotic Studies of Novel 1,3,4-Oxadiazole Derivatives
Source: Int J Mol Sci. 2018 Nov 15;19(11):3606. doi: 10.3390/ijms19113606 (PMC6274789; doi:10.3390/ijms19113606)
Supplement: Supplementary file 1 [file ijms-19-03606-s001.pdf]

International Journal of
Molecular Sciences

MDPI

Molecular Docking, Computational, and Antithrombotic Studies of Novel 1,3,4-Oxadiazole Derivatives

Majda Batool [1], Affifa Tajammal [1], Firdous Farhat [1], Francis Verpoort [2], Zafar A.K. Khattak [2], Mehr-un-Nisa [1,3], Muhammad Shahid [1], Hafiz Adnan Ahmad [1,4], Munawar Ali Munawar [1,*], Muhammad Zia-ur-Rehman [5,*] and Muhammad Asim Raza Basra [1,*]

1 Institute of Chemistry, University of the Punjab, New Campus, Lahore 54590, Pakistan; majdabatool12@gmail.com (M.B.); affifa.tajammal@yahoo.com (A.T.); farhatfirdous555@gmail.com (F.F.); anmehrunnisa@gmail.com (M.N.); mshahidkhan403@gmail.com (M.S.); adnan.ahmad.pu@hotmail.com (H.A.A.)

2 Laboratory of Organometallics, Catalysis and Ordered Materials, State Key Laboratory of Advanced Technology for Material Synthesis and Processing, Wuhan University of Technology, Wuhan 430070, China; Francis.Verpoort@ghent.ac.kr (F.V.); zafarchem_qau@yahoo.com (Z.A.K.K.)

3 Division of Science & Technology, University of Education, Township, Lahore 54770, Pakistan

4 Key Laboratory of Synthetic and Nature Functional Molecule Chemistry of Ministry of Education, Department of Chemistry & Materials Science, Northwest University, Xi'an 710127, China

5 Applied Chemistry Research Centre, PCSIR Laboratories Complex, Ferozpur Road, Lahore 54600, Pakistan

* Correspondence: munawaralimunawar@yahoo.com (M.A.M.); rehman_pcsir@hotmail.com (M.Z.R.); asimbasra@gmail.com (M.A.R.B.); Tel.: +92-332-4392363 (M.A.M.); Tel.: +92-333-3451977 (M.Z.R.); Tel.: +92-42-9923046 Ext.836 (M.A.R.B.)

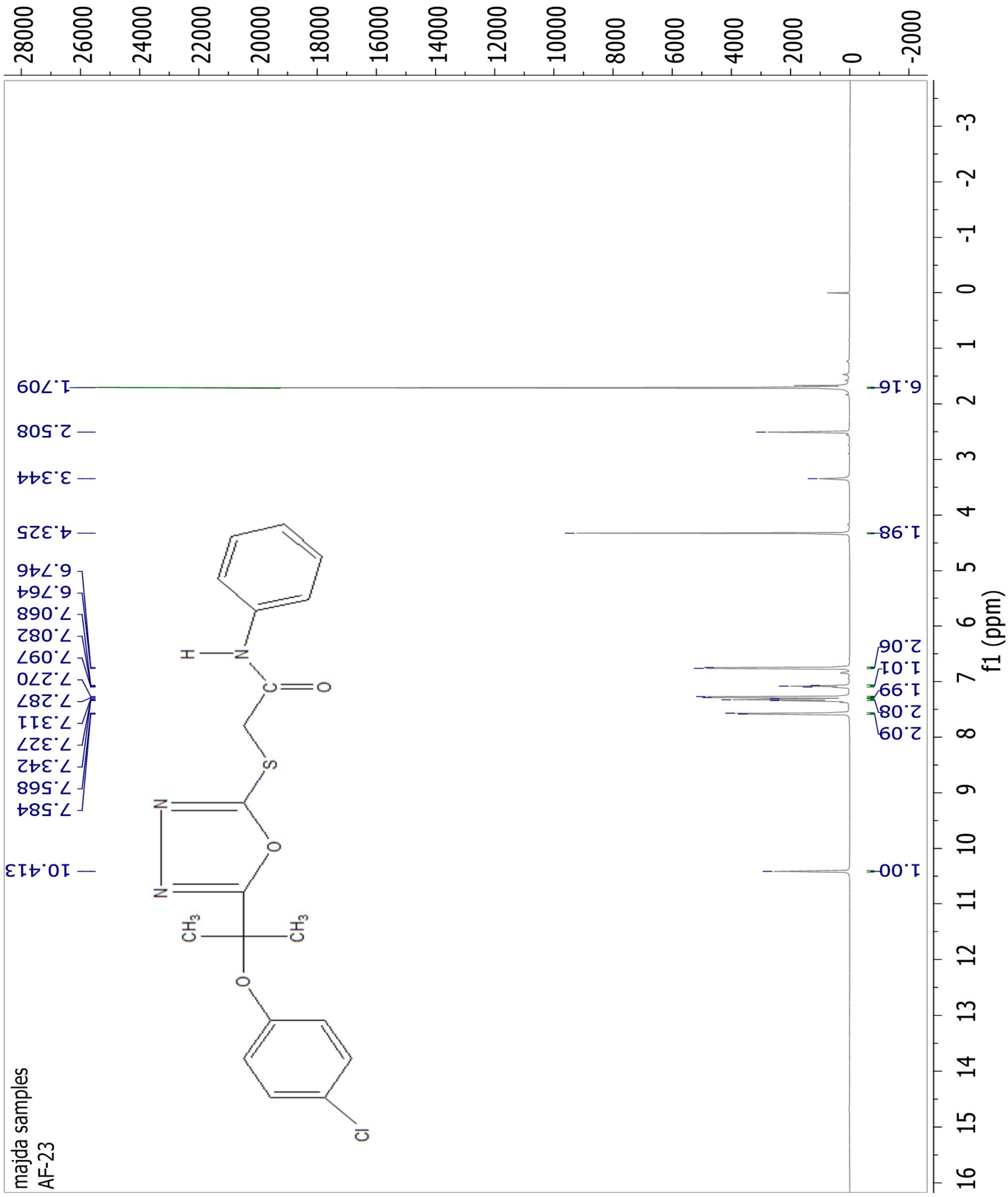

Figure S1. ^{1}H-NMR spectrum of compound 3a.

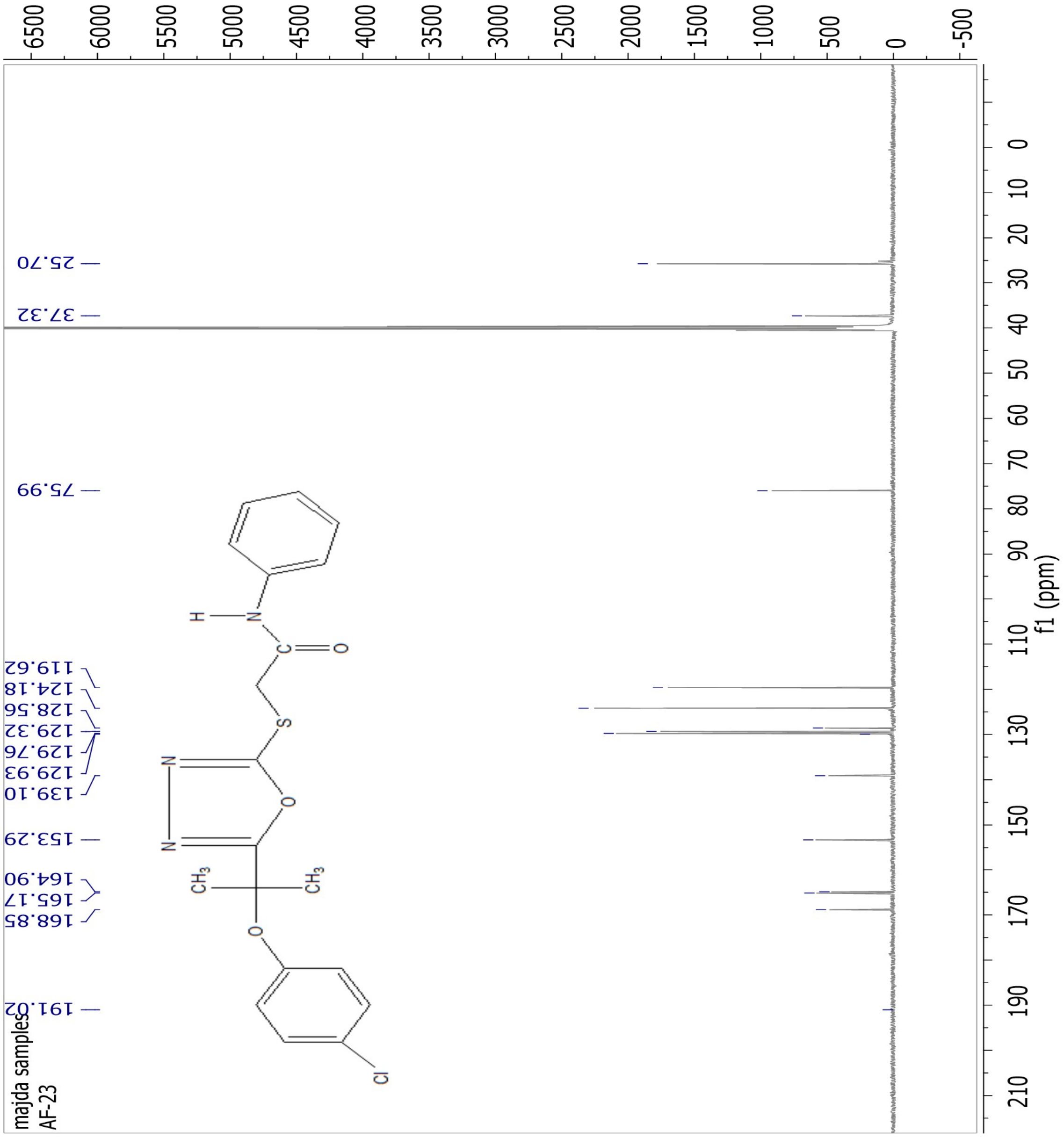

Figure S2. ^{13}C-NMR spectrum of compound 3a.

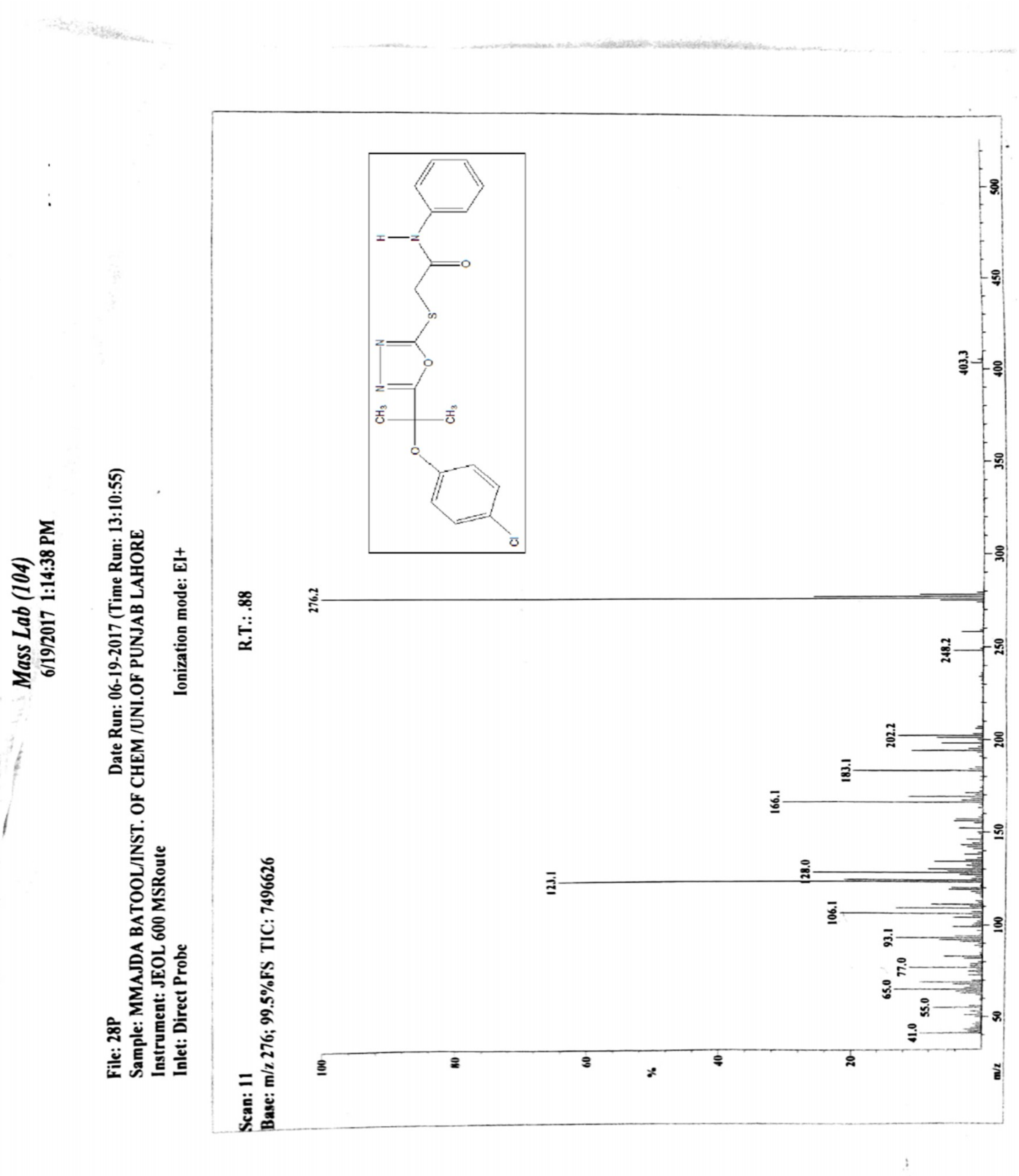

Figure S3. EI-MS of compound 3a.

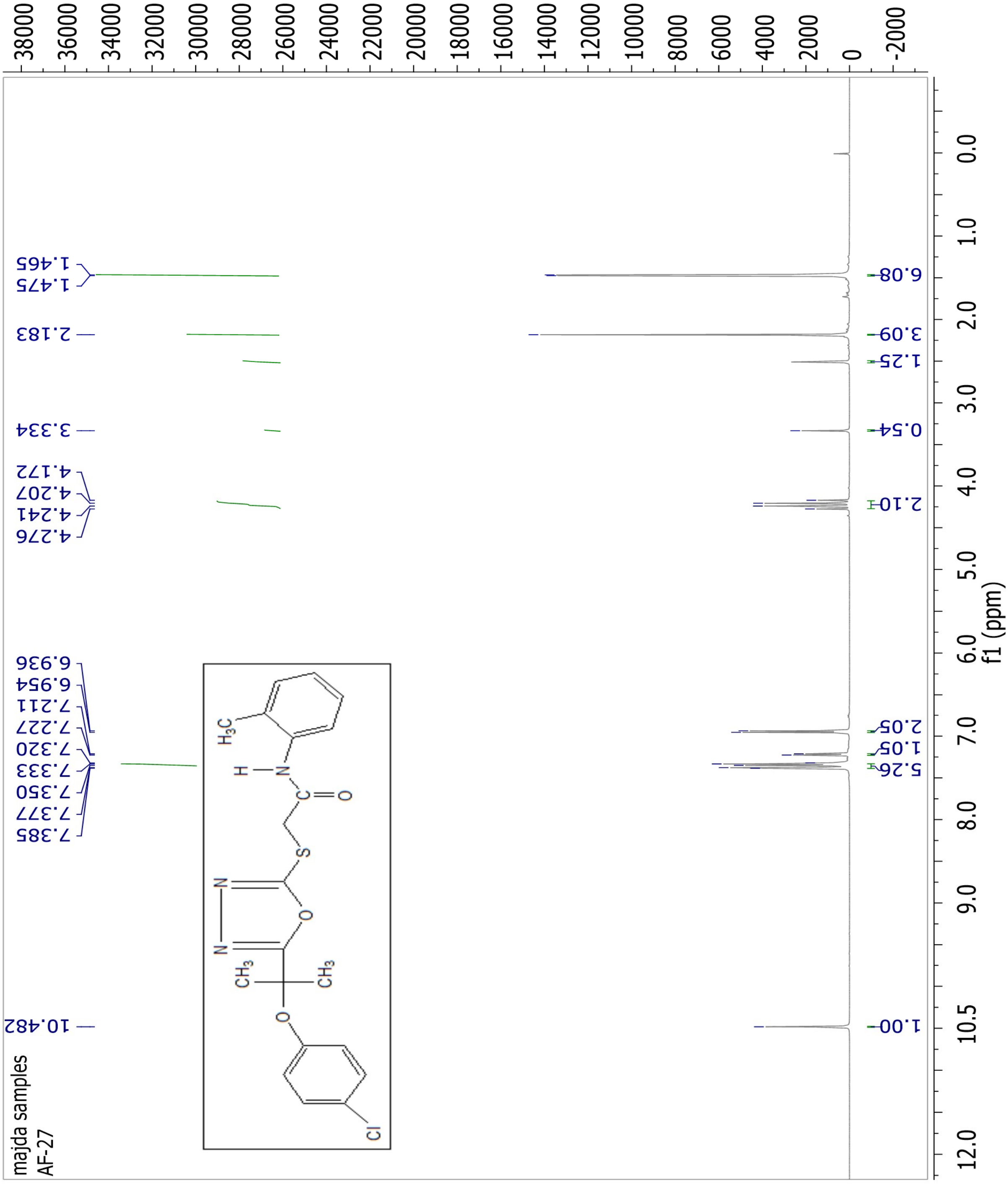

Figure S4. ^{1}H-NMR spectrum of compound 3b.

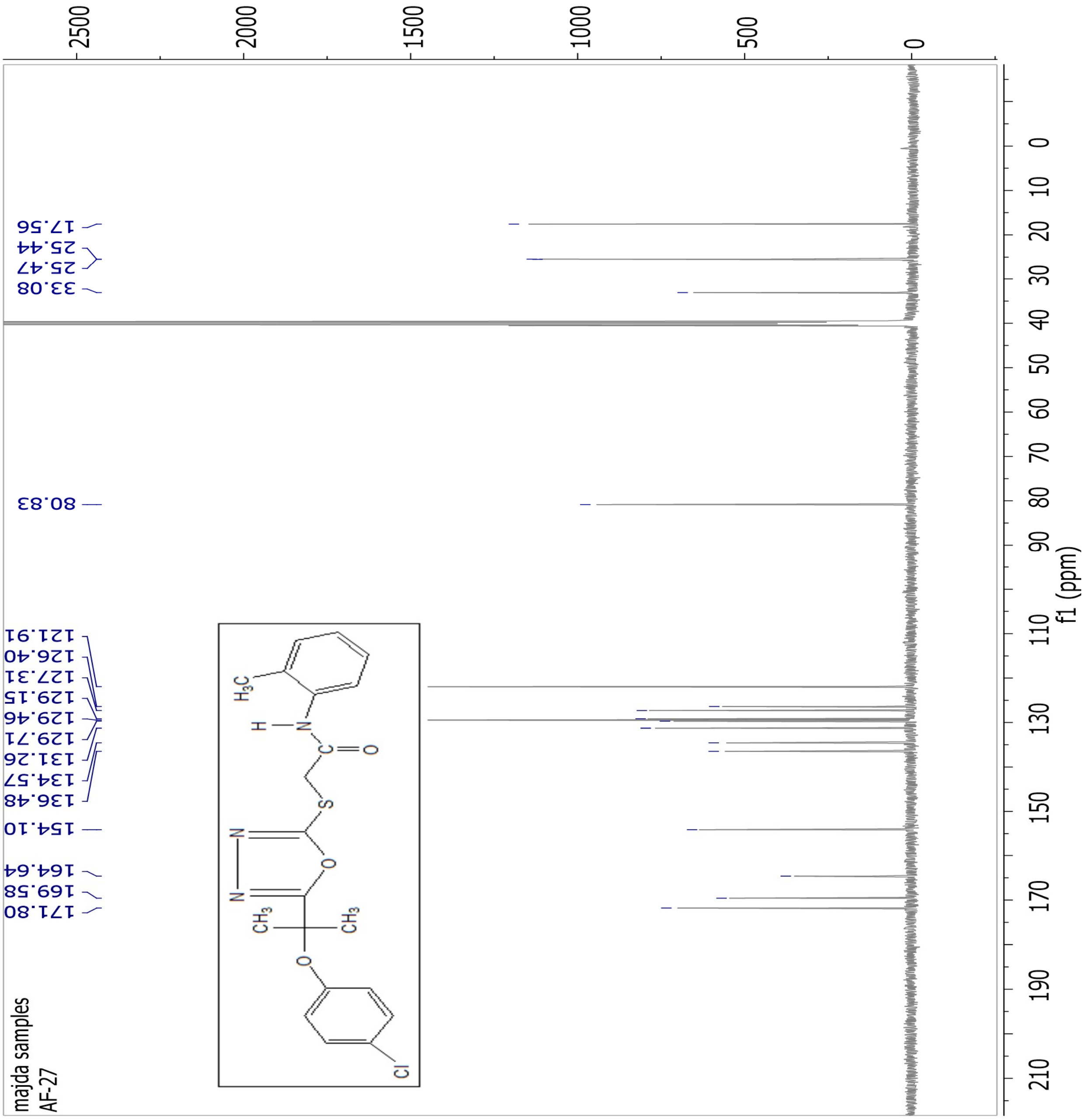

Figure S5. ^{13}C-NMR spectrum of compound 3b.

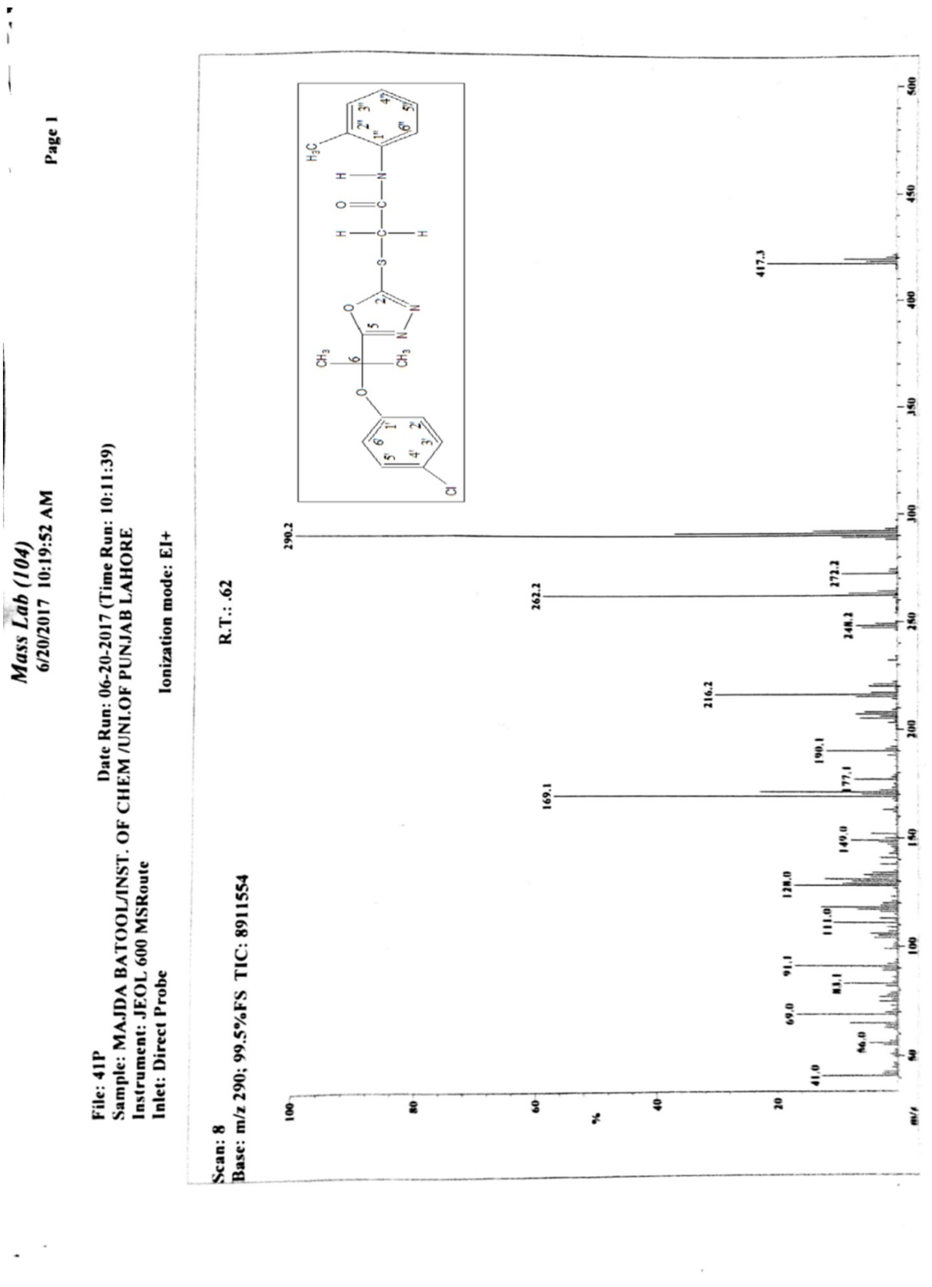

Figure S6. EI-MS of compound 3b.

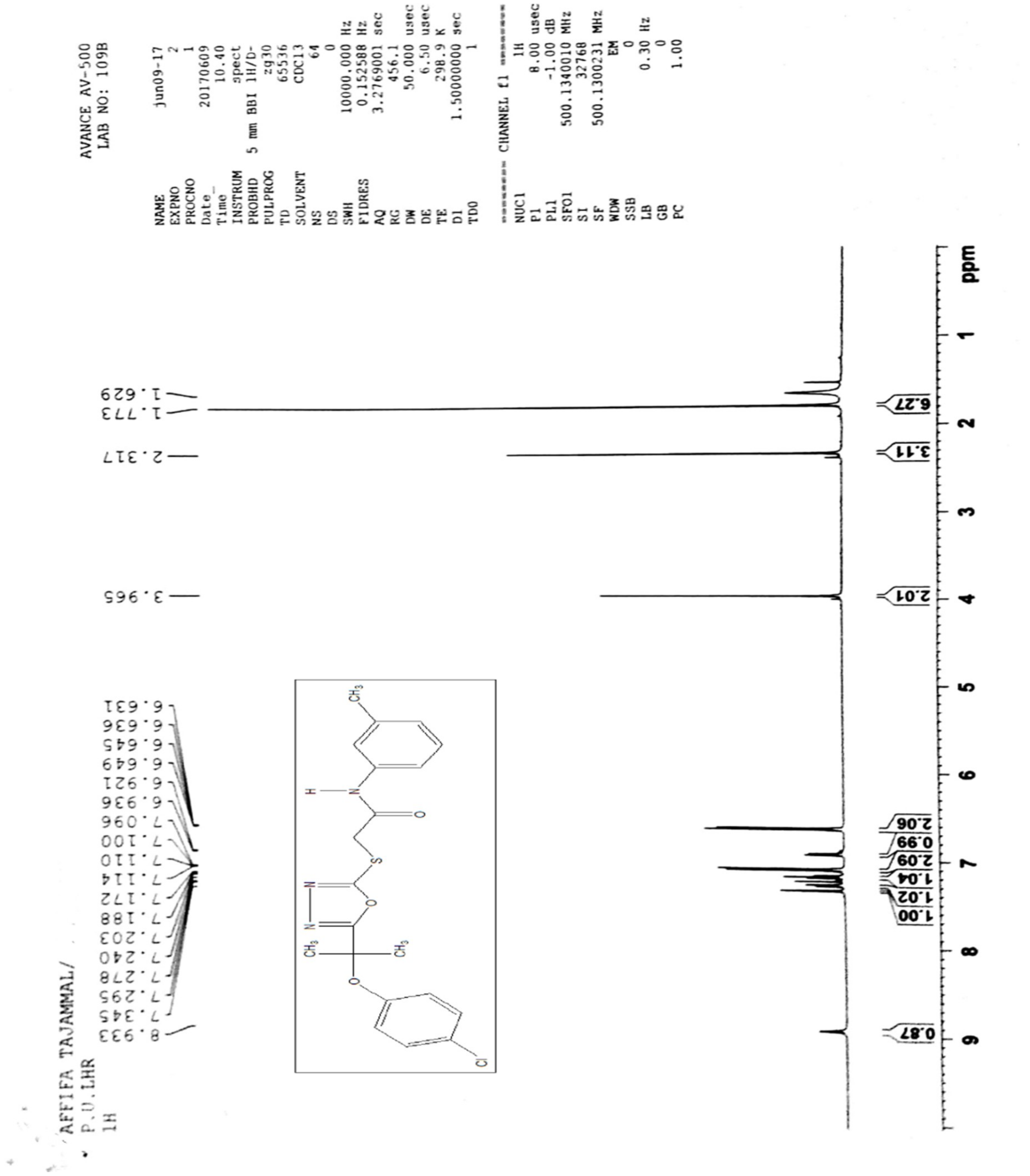

Figure S7. ^{1}H-NMR spectrum of compound 3c.

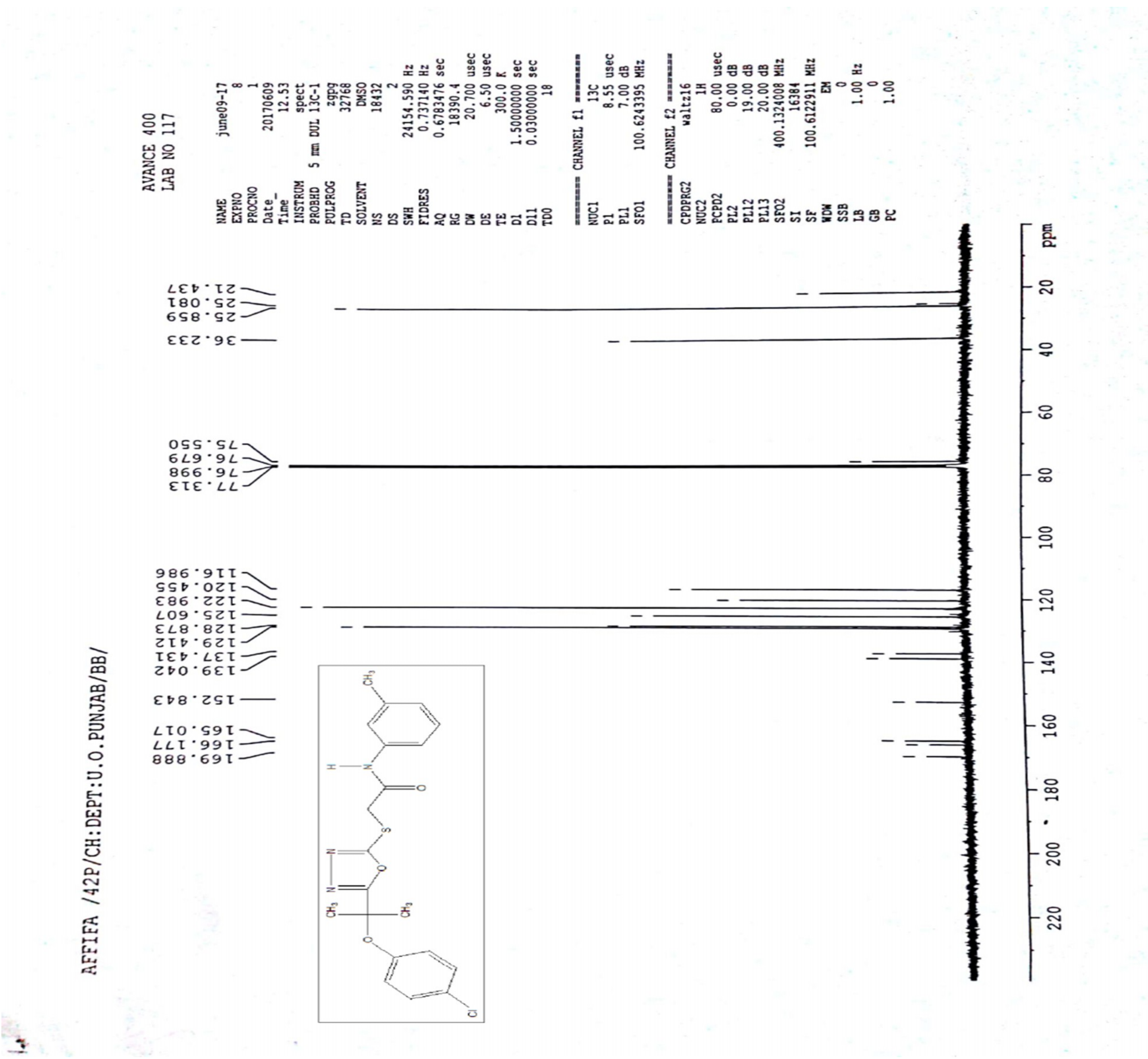

Figure S8. ^{13}C-NMR spectrum of compound 3c.

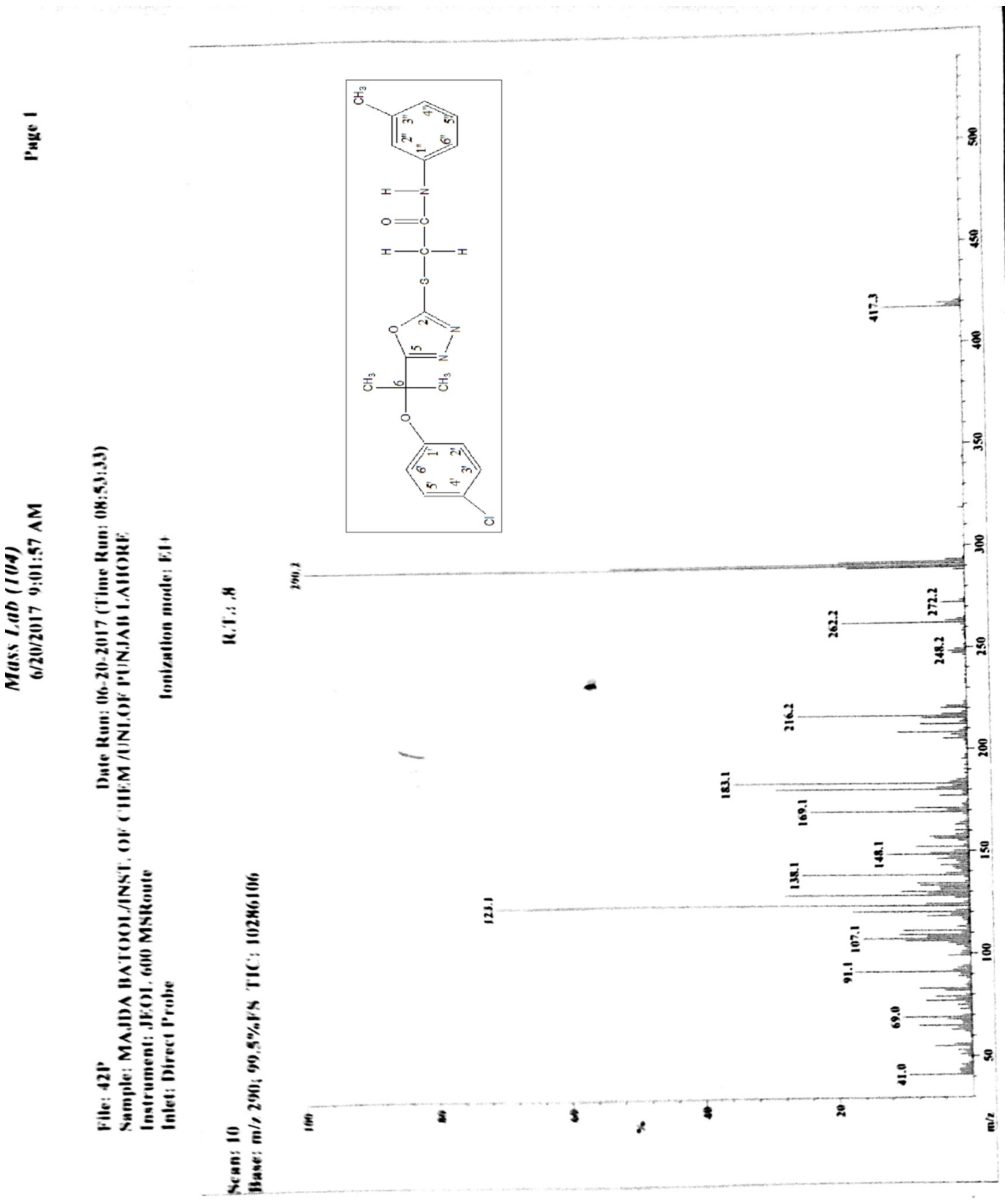

Figure S9. EI-MS of compound 3c.

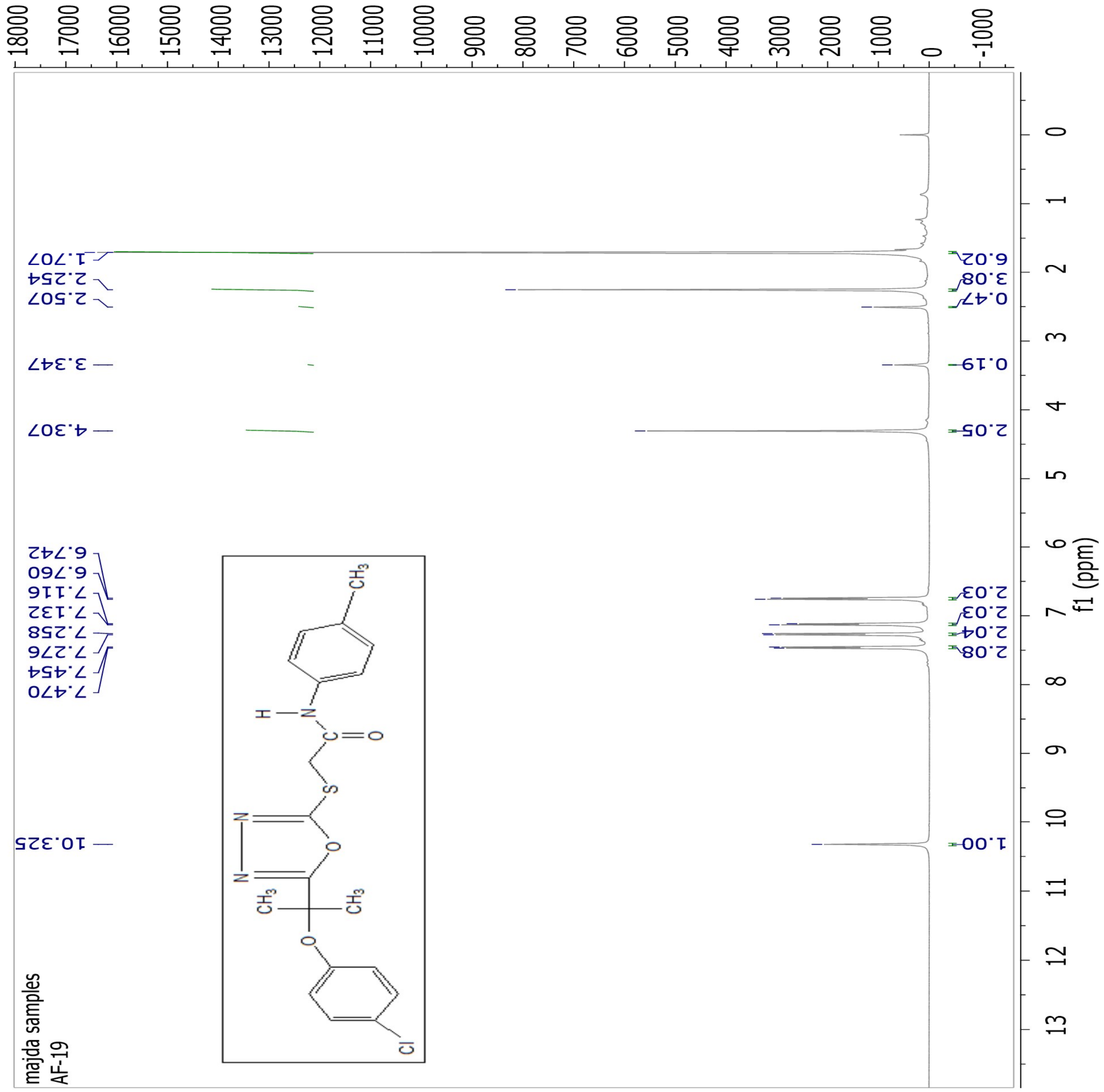

Figure S10. ^{1}H-NMR spectrum of compound 3d.

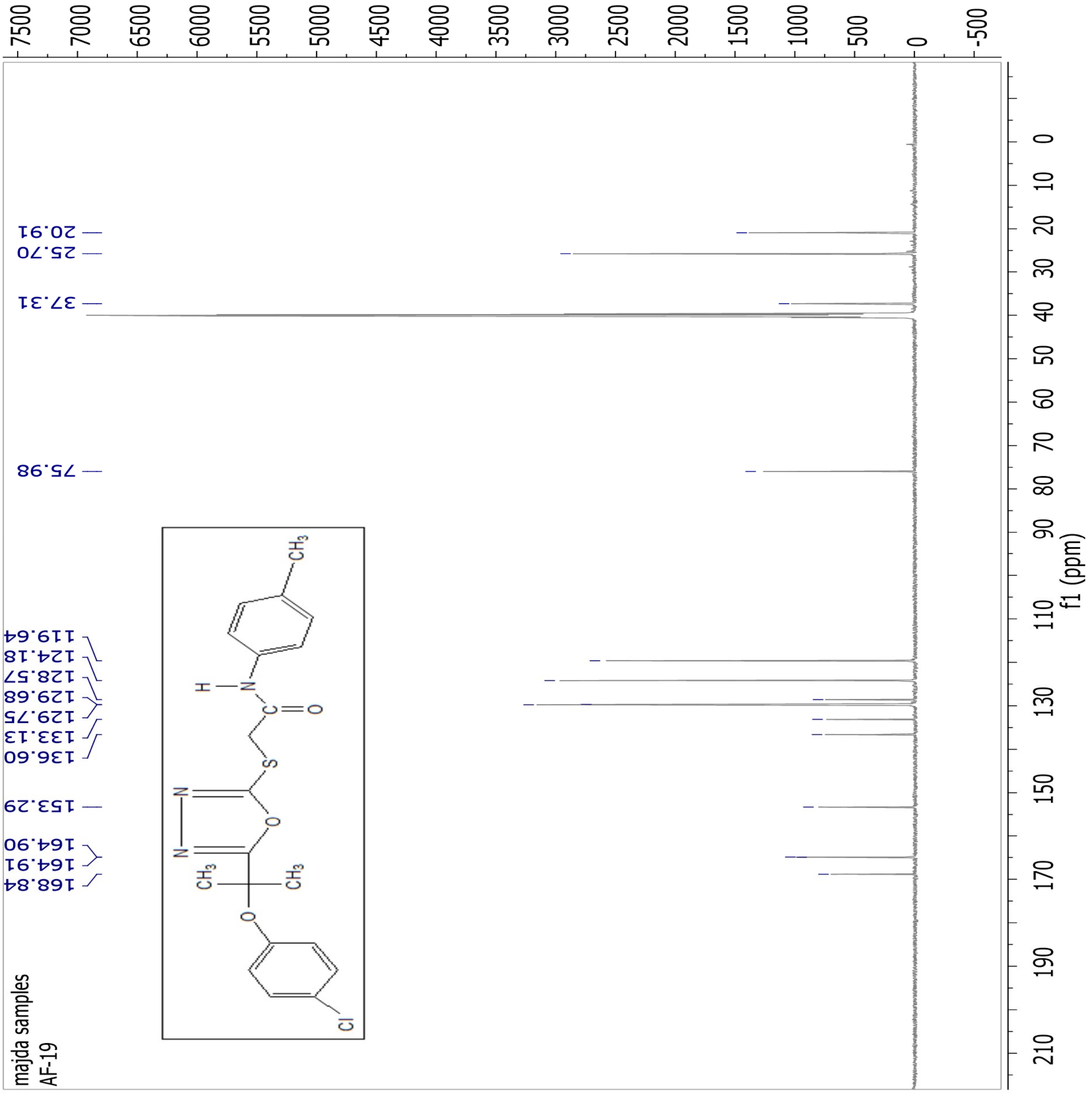

Figure S11. ^{13}C-NMR spectrums of compound 3d.

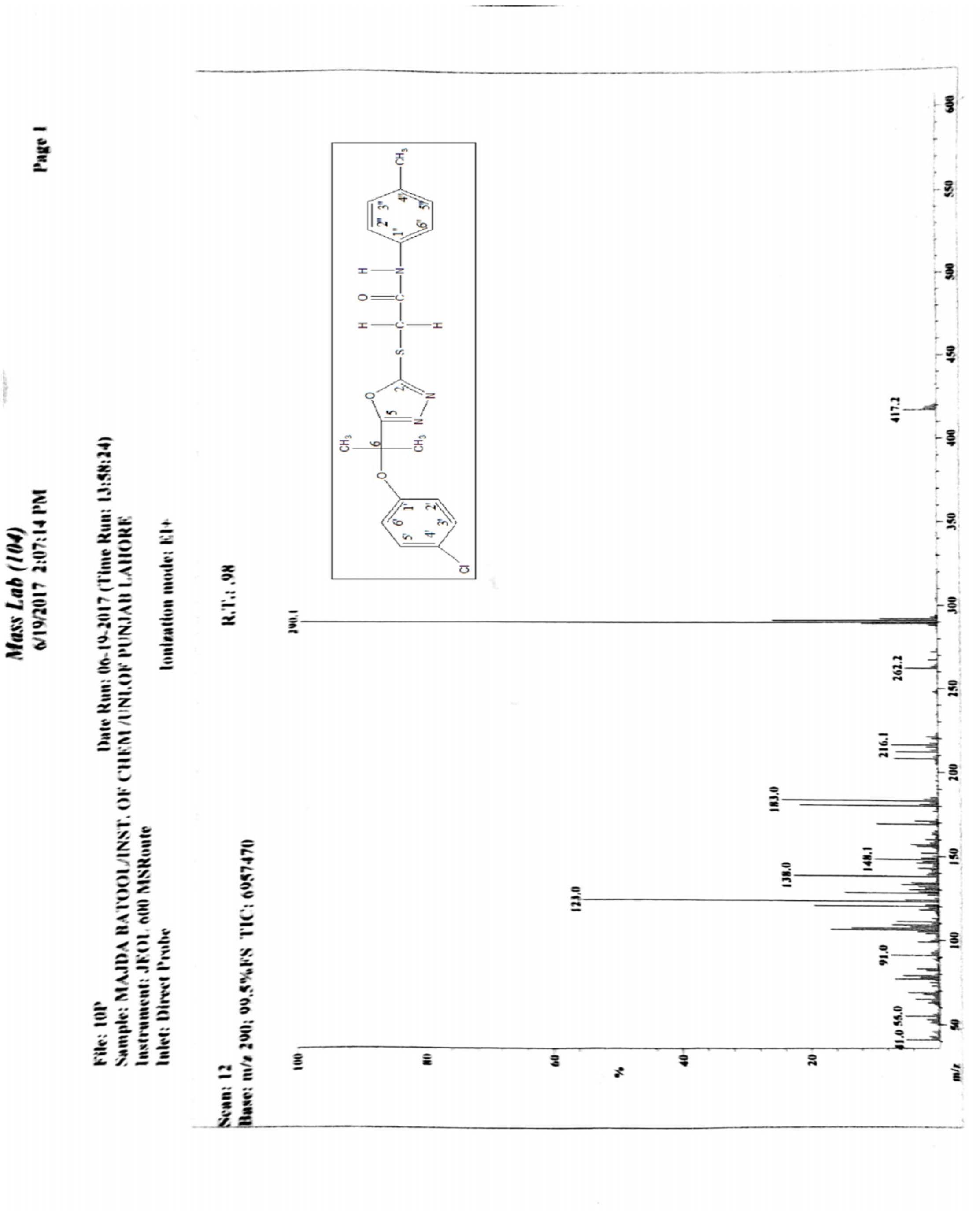

Figure S12. EI-MS of compound 3d.

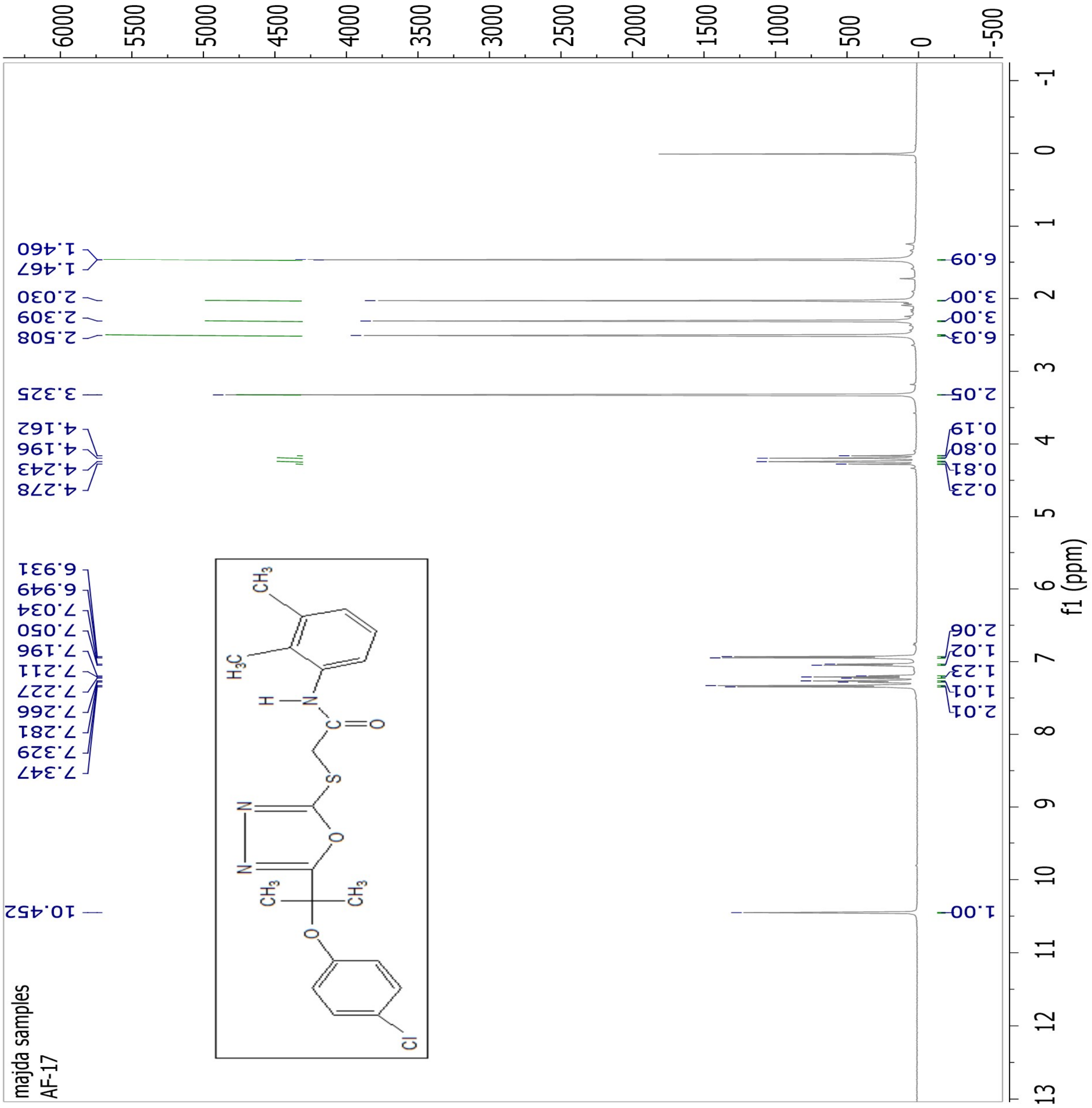

Figure S13. ^{1}H-NMR spectrum of compound 3e.

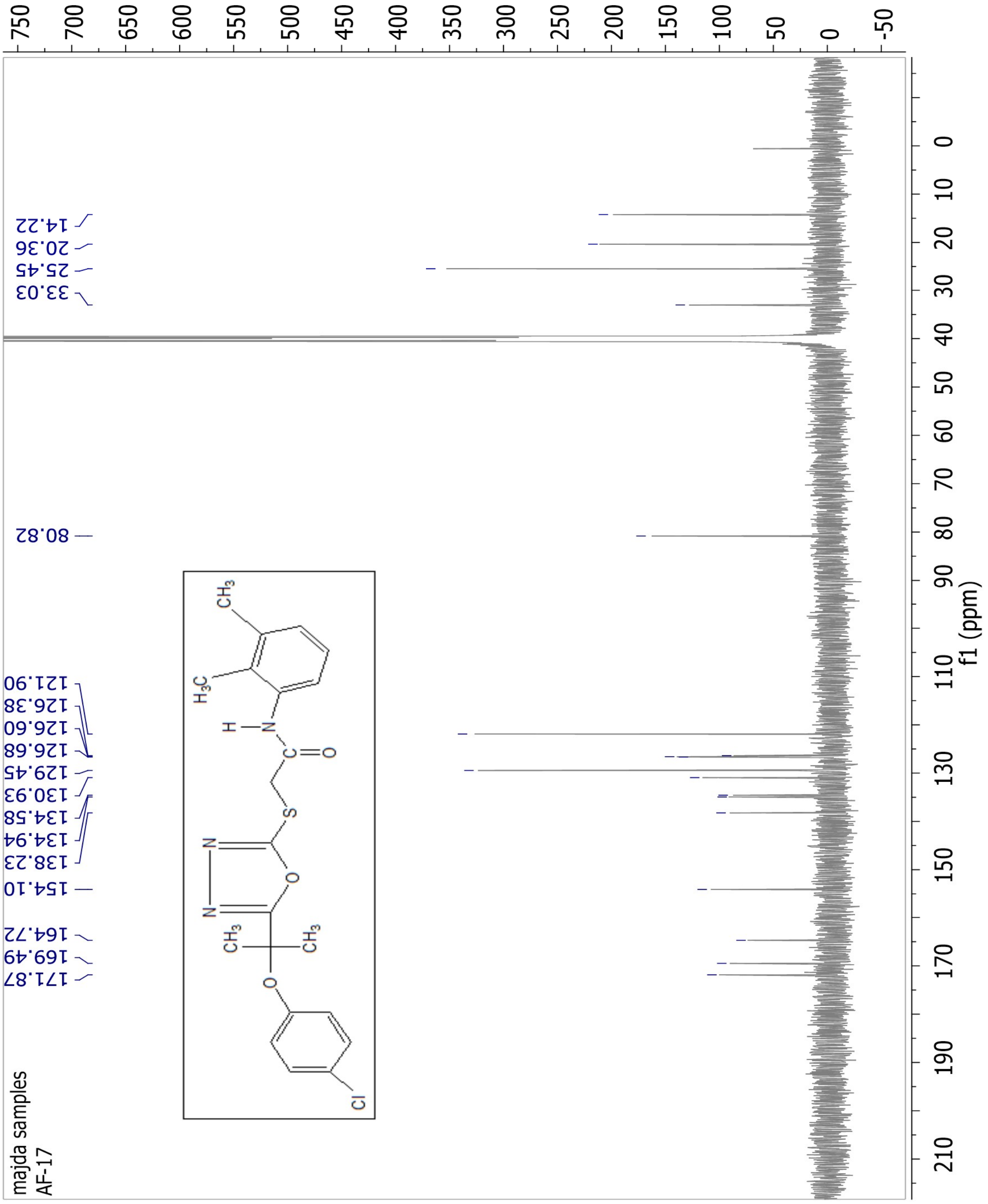

Figure S14. ^{13}C-NMR spectrum of compound 3e.

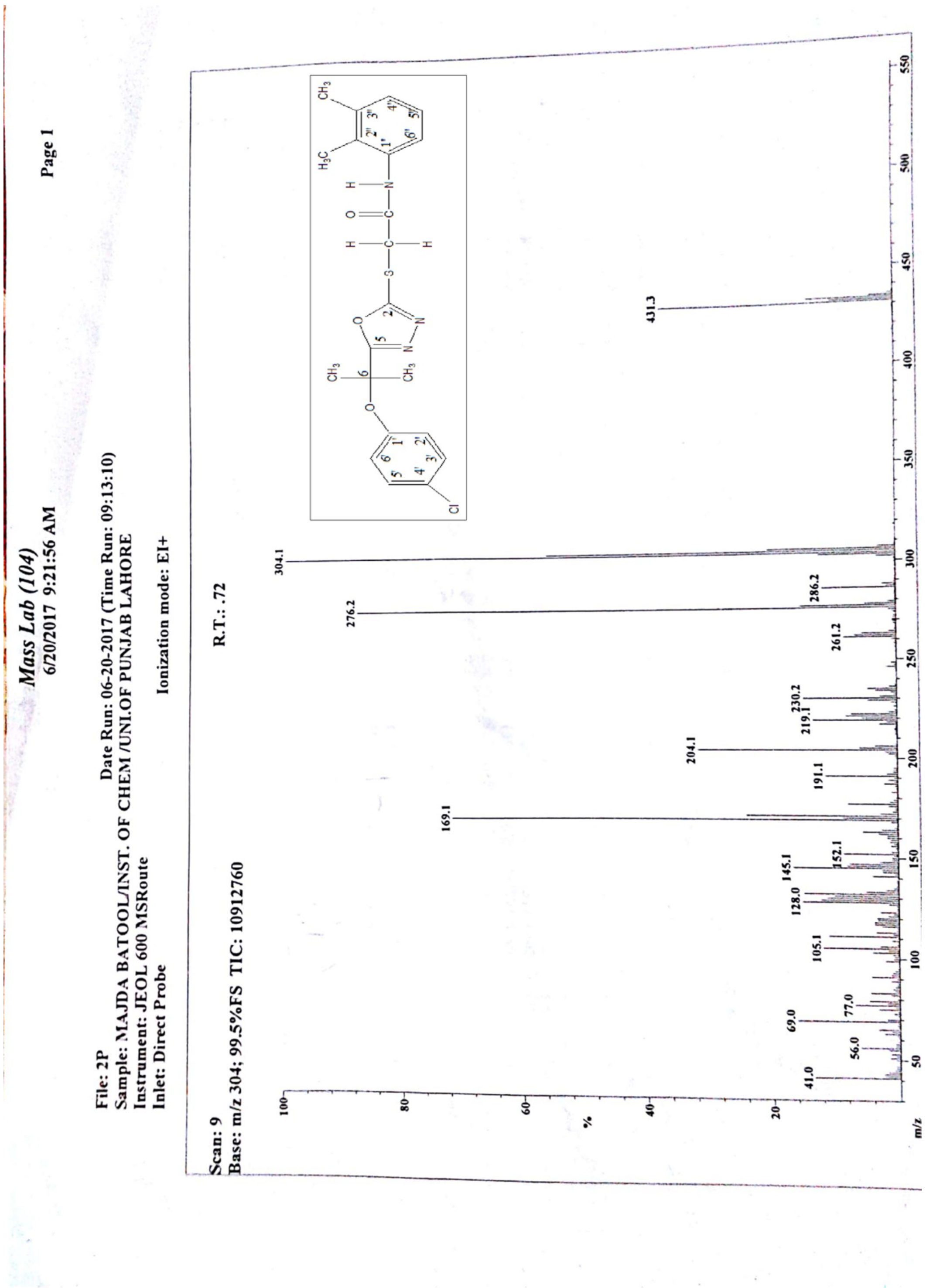

Figure S15. EI-MS of compound 3e.

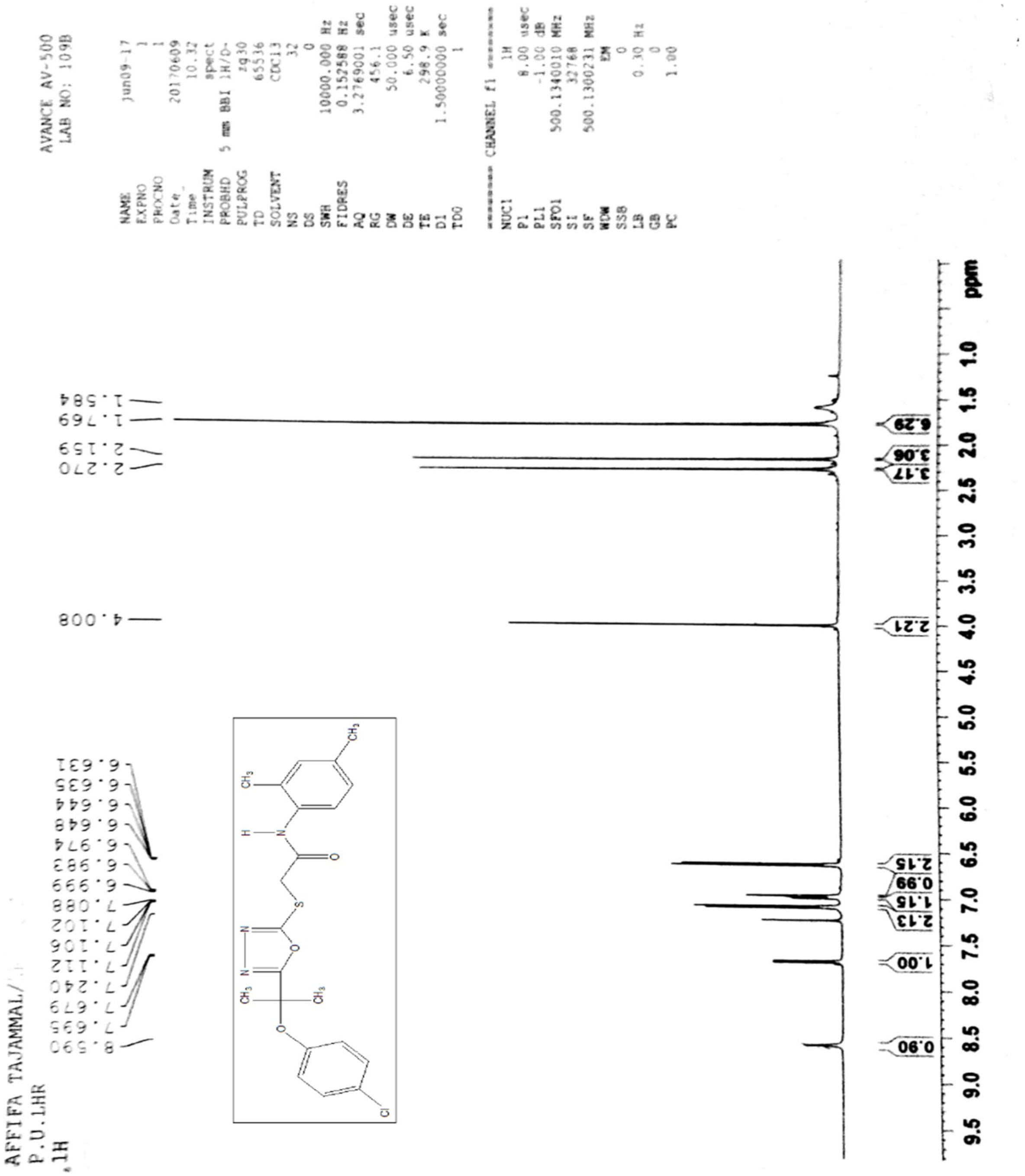

Figure S16. ^{1}H-NMR spectrum of compound 3f.

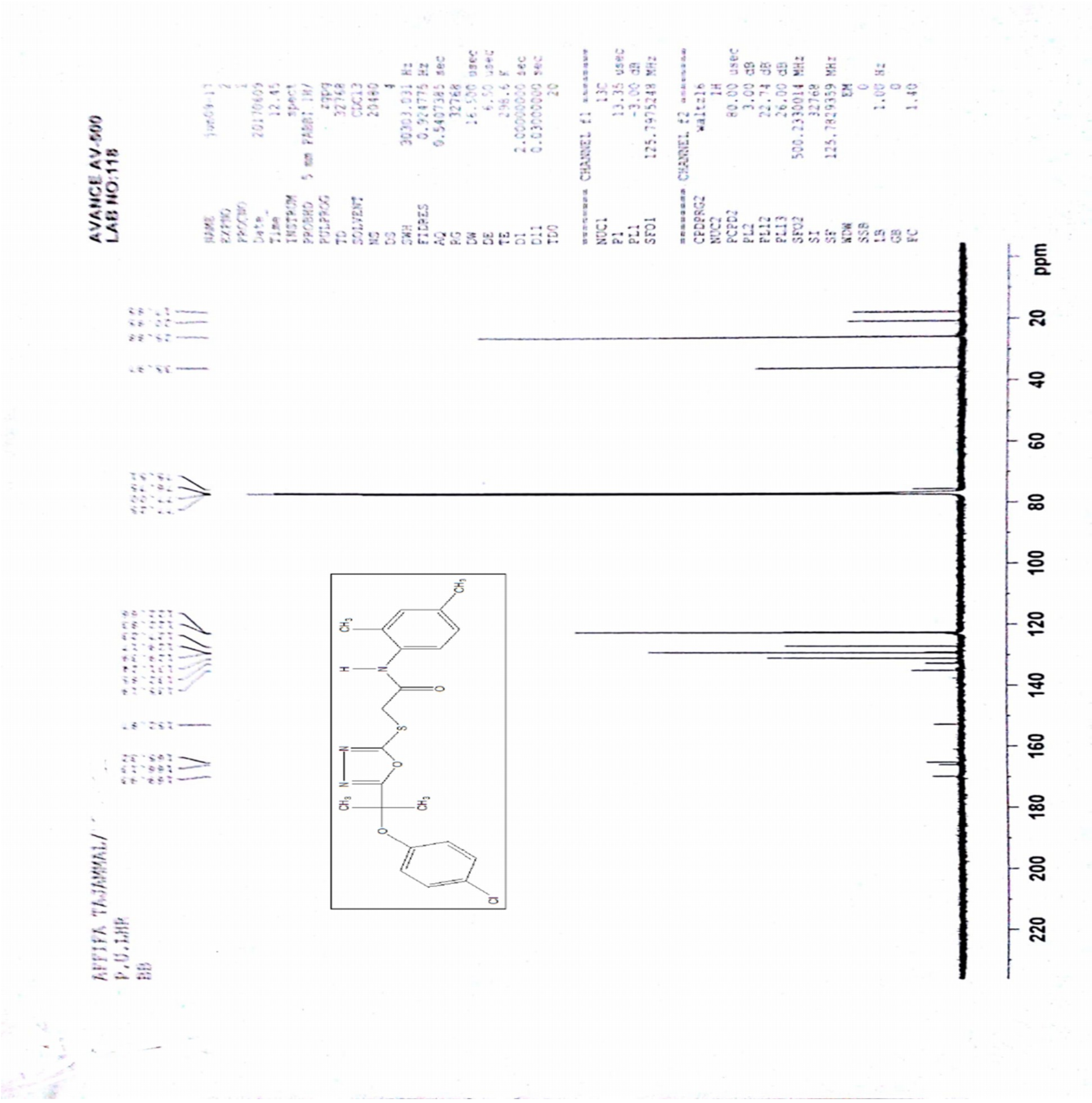

Figure S17. ^{13}C-NMR spectrum of compound 3f.

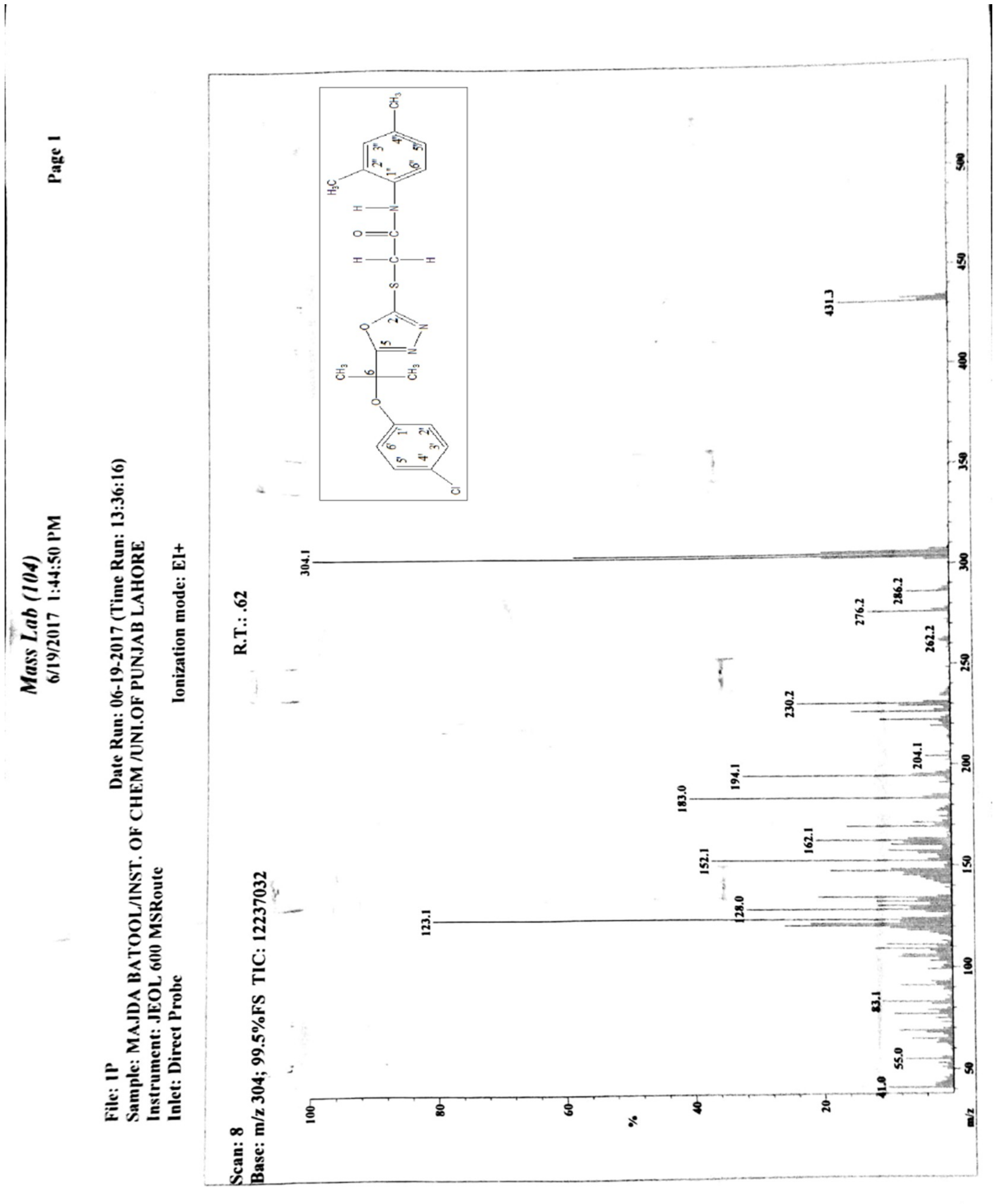

Figure S18. EI-MS of compound 3f.

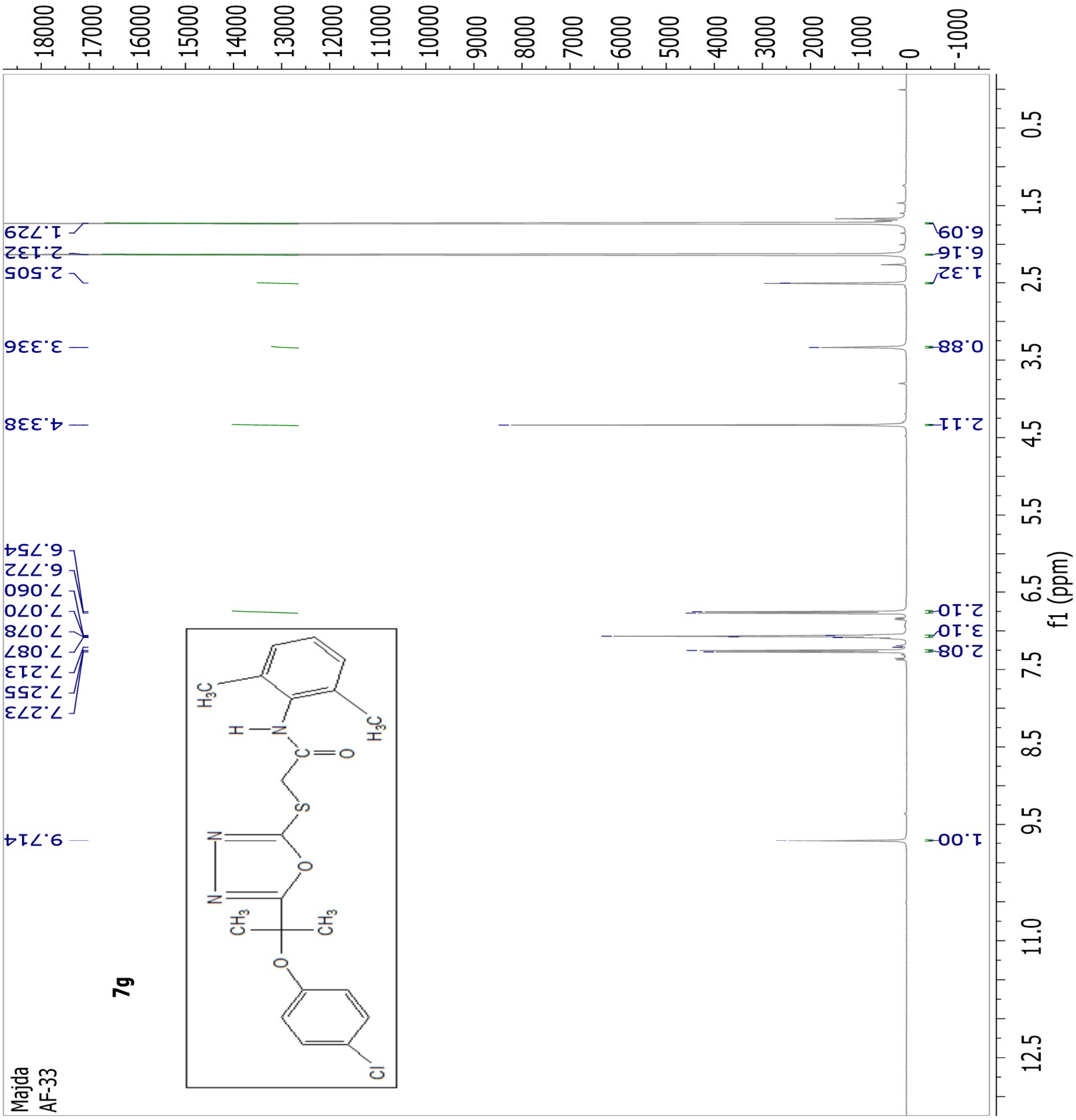

Figure S19. ^{1}H-NMR spectrum of compound 3g.

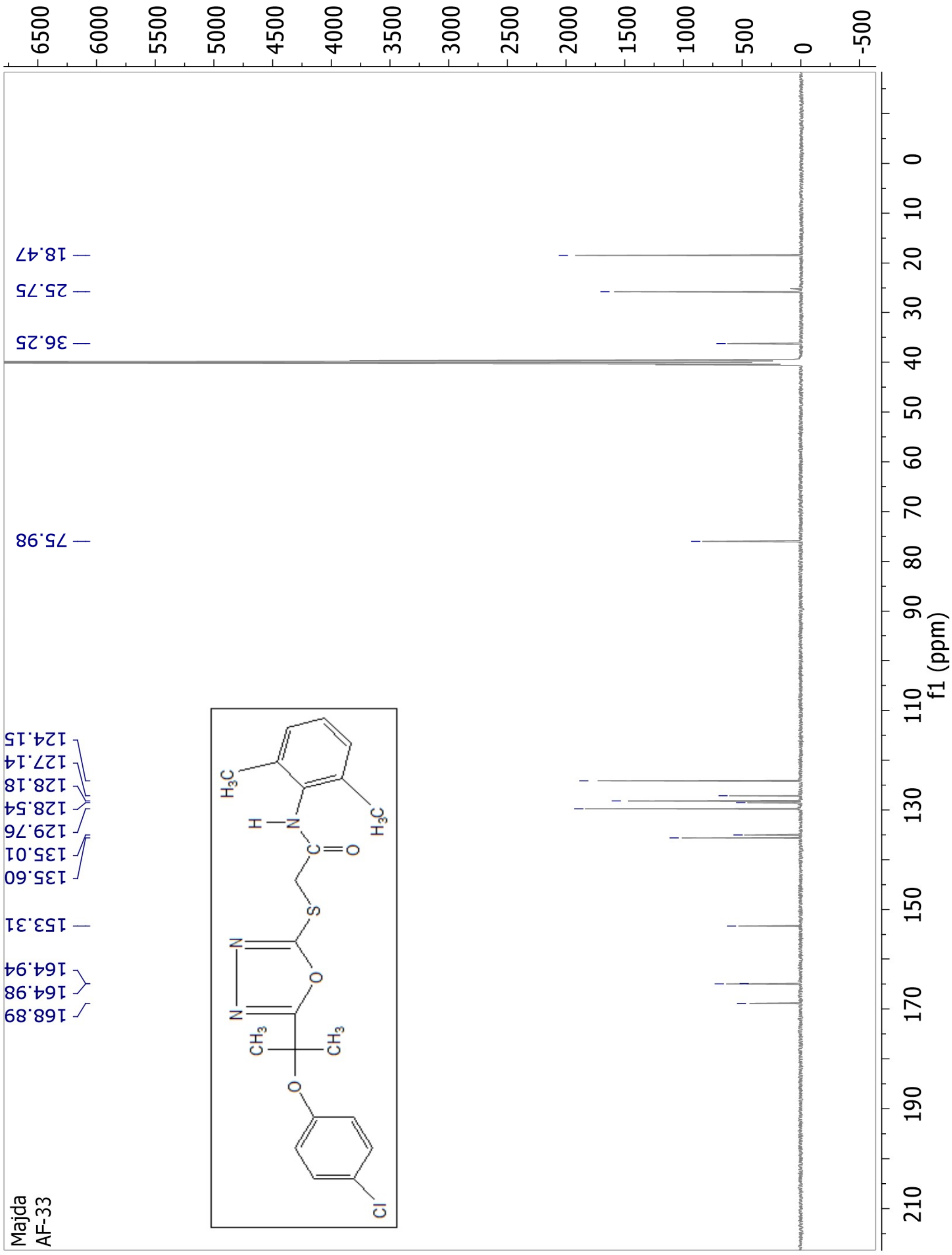

Figure S20. ^{13}C-NMR spectrum of compound 3g.

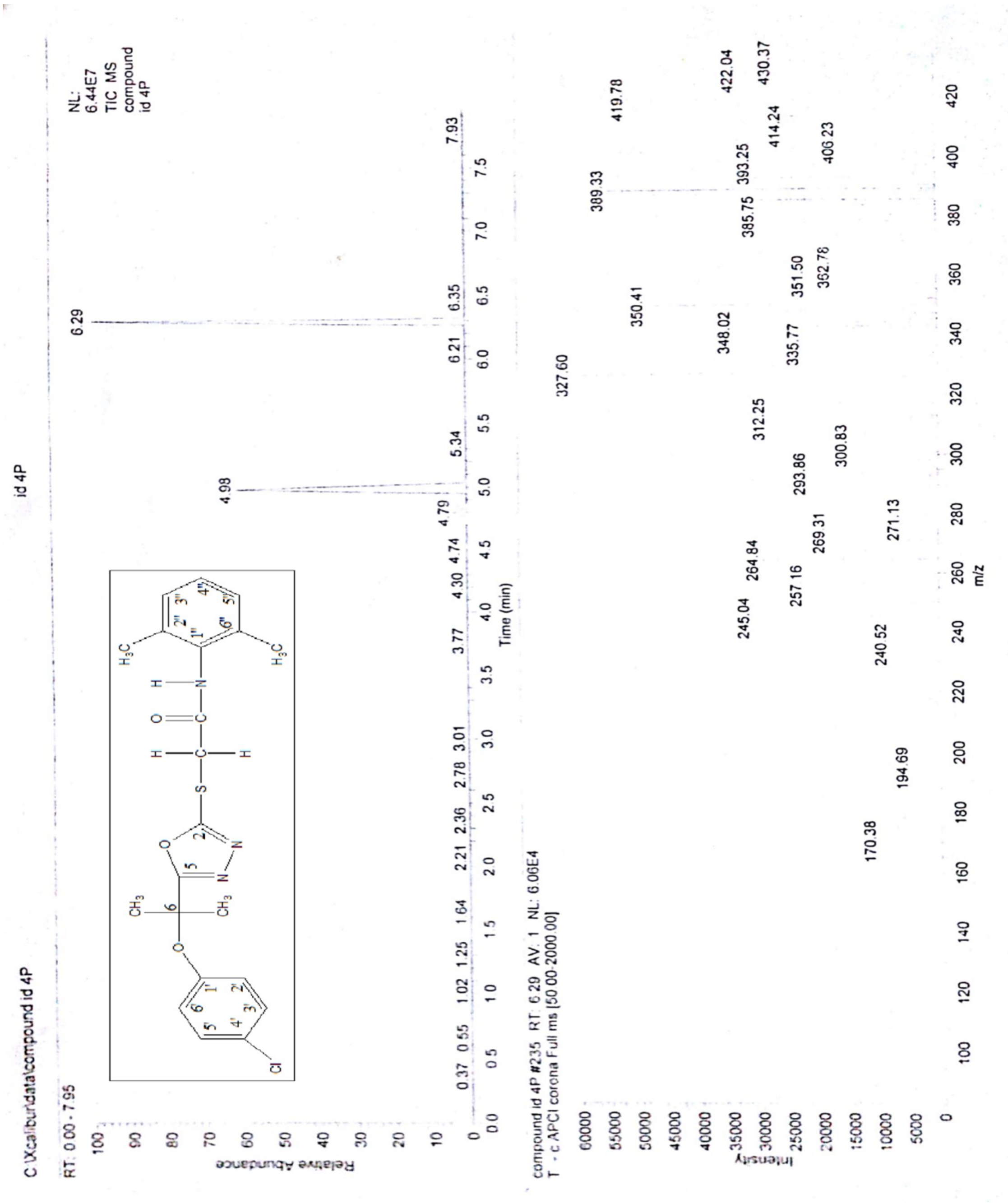

Figure S21. EI-MS of compound 3g.

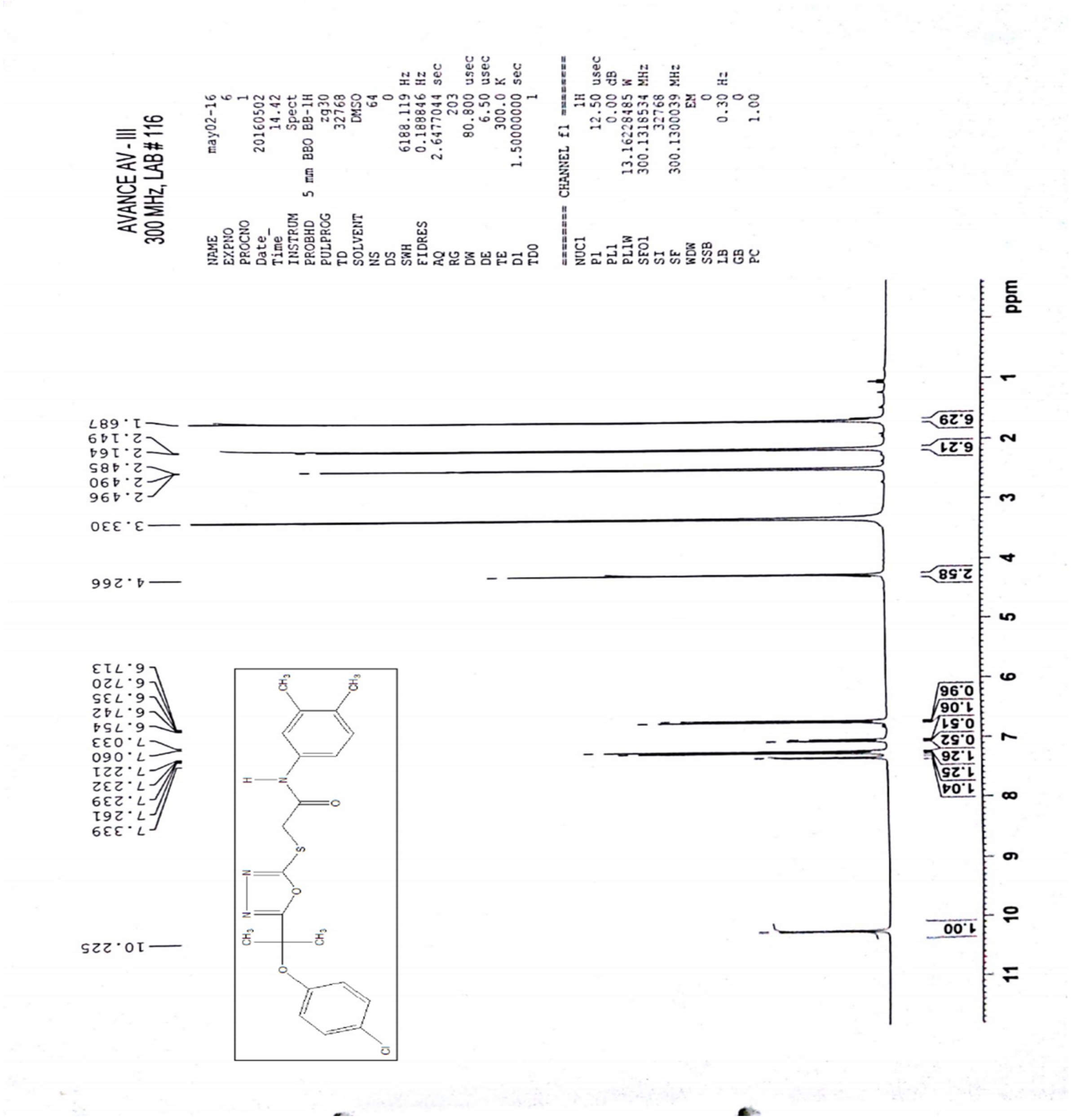

Figure S22. ¹H-NMR spectrum of compound 3h.

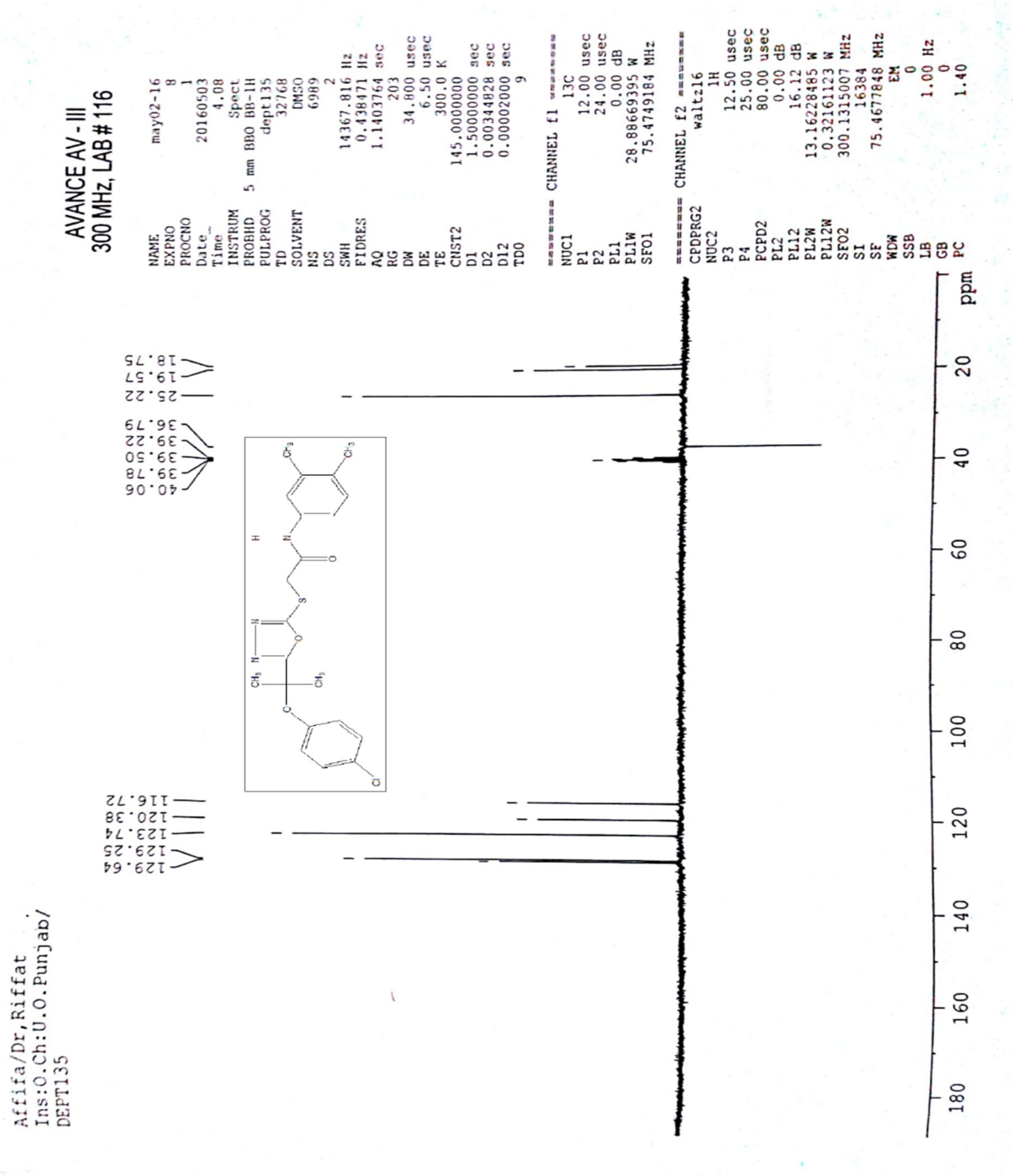

Figure S23. ^{13}C-NMR spectrum of compound 3h.

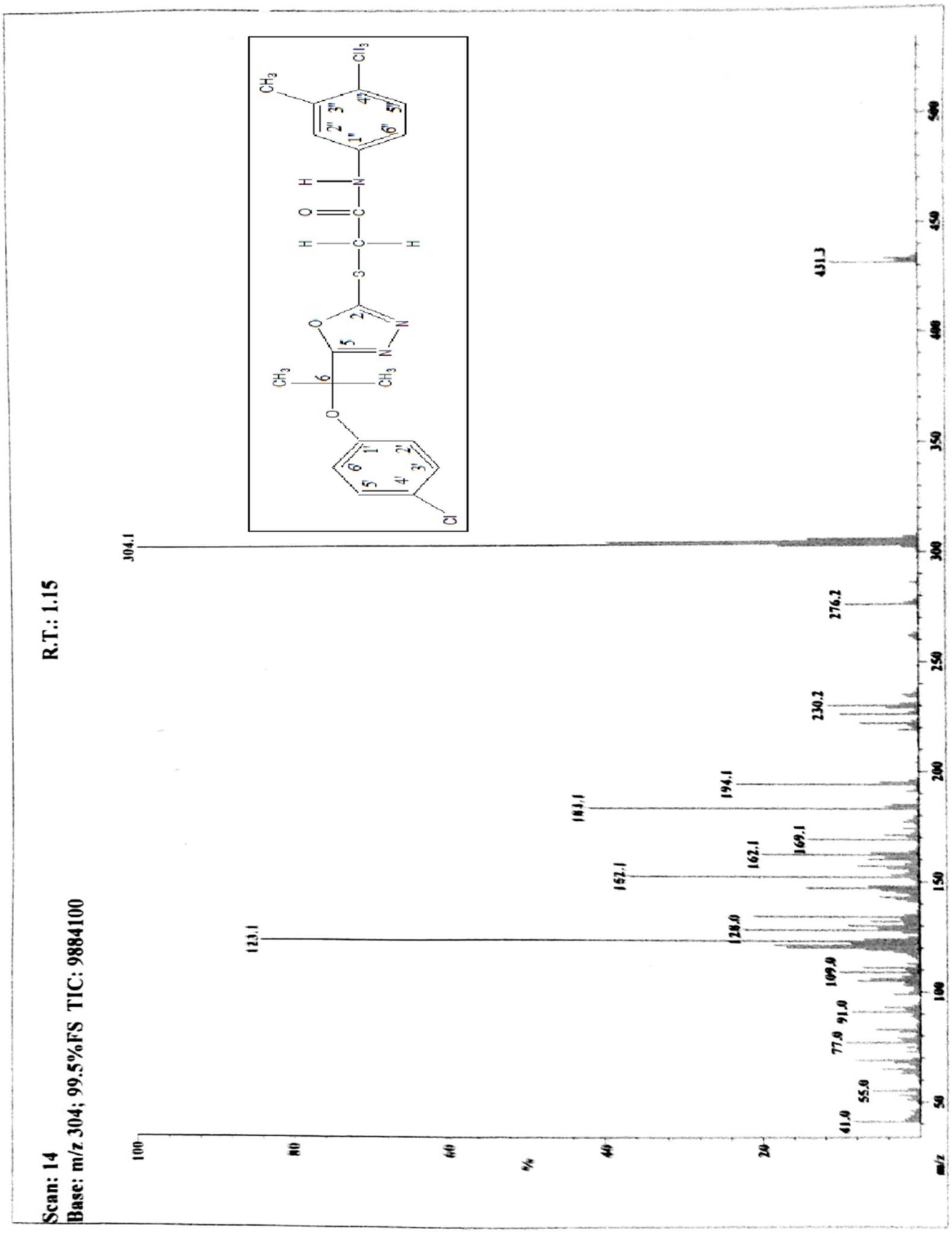

Figure S24. EI-MS of compound 3h.

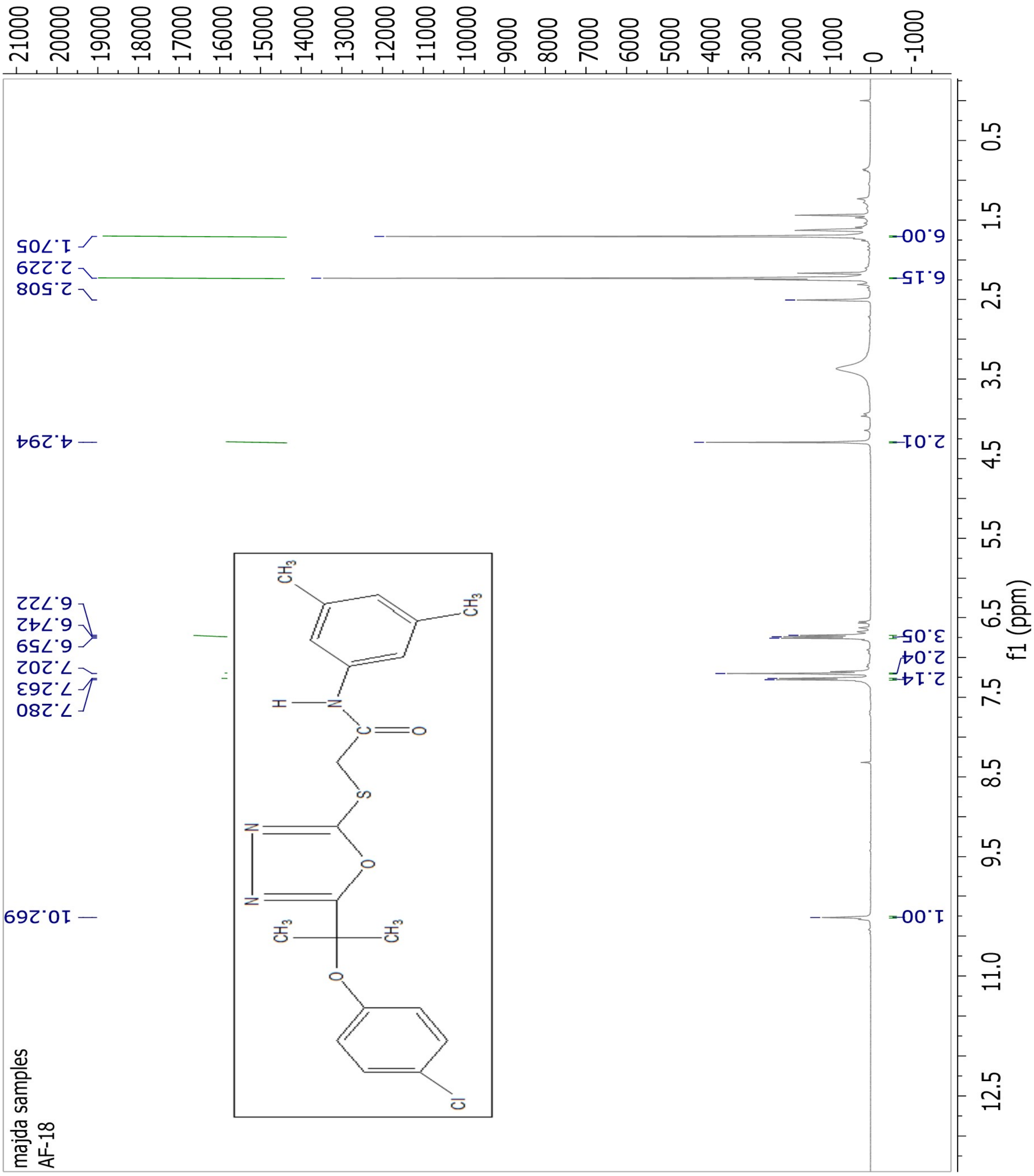

Figure S25. ^{1}H-NMR spectrum of compound 3i.

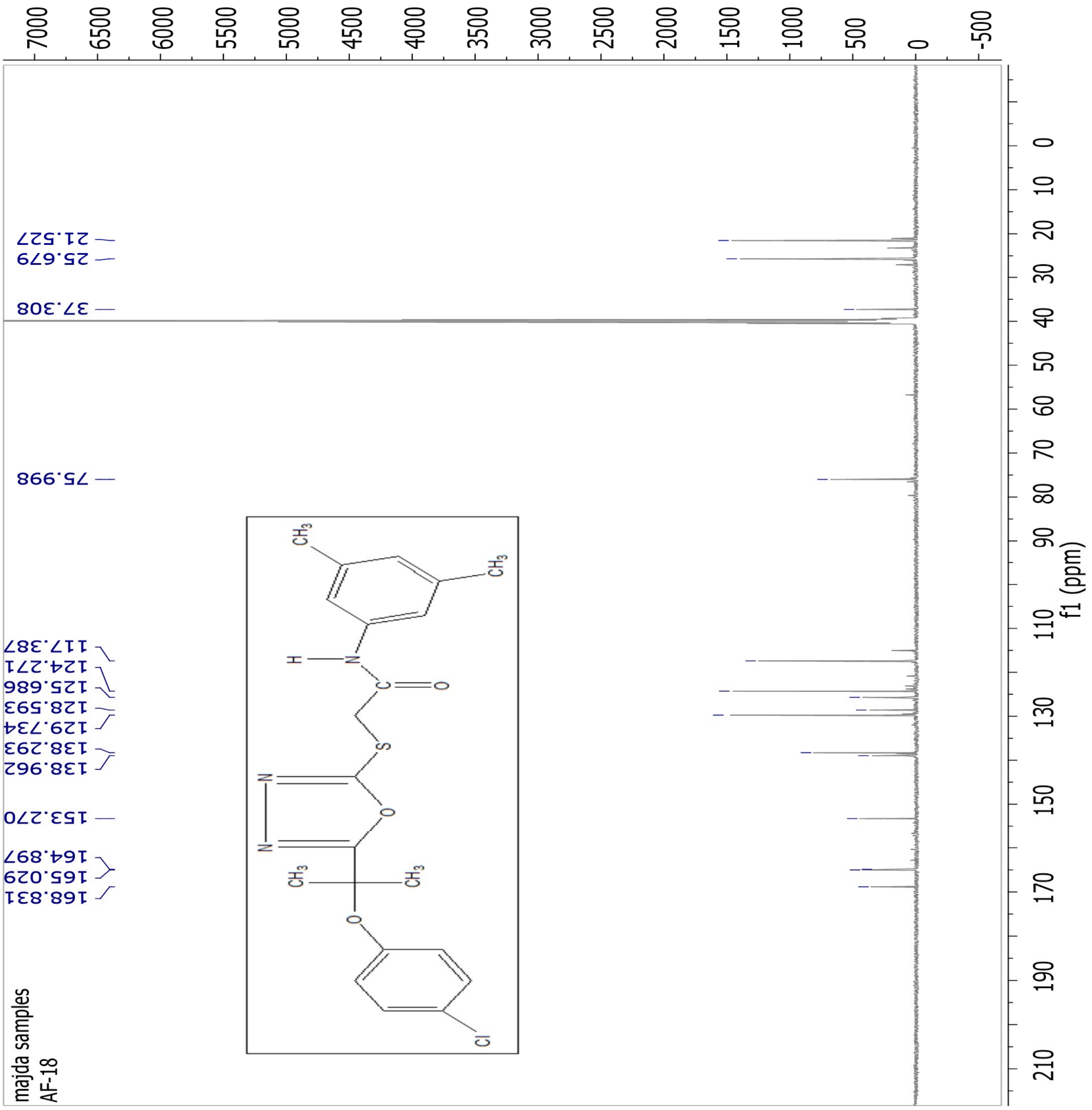

Figure S26. ^{13}C-NMR spectrum of compound 3i.

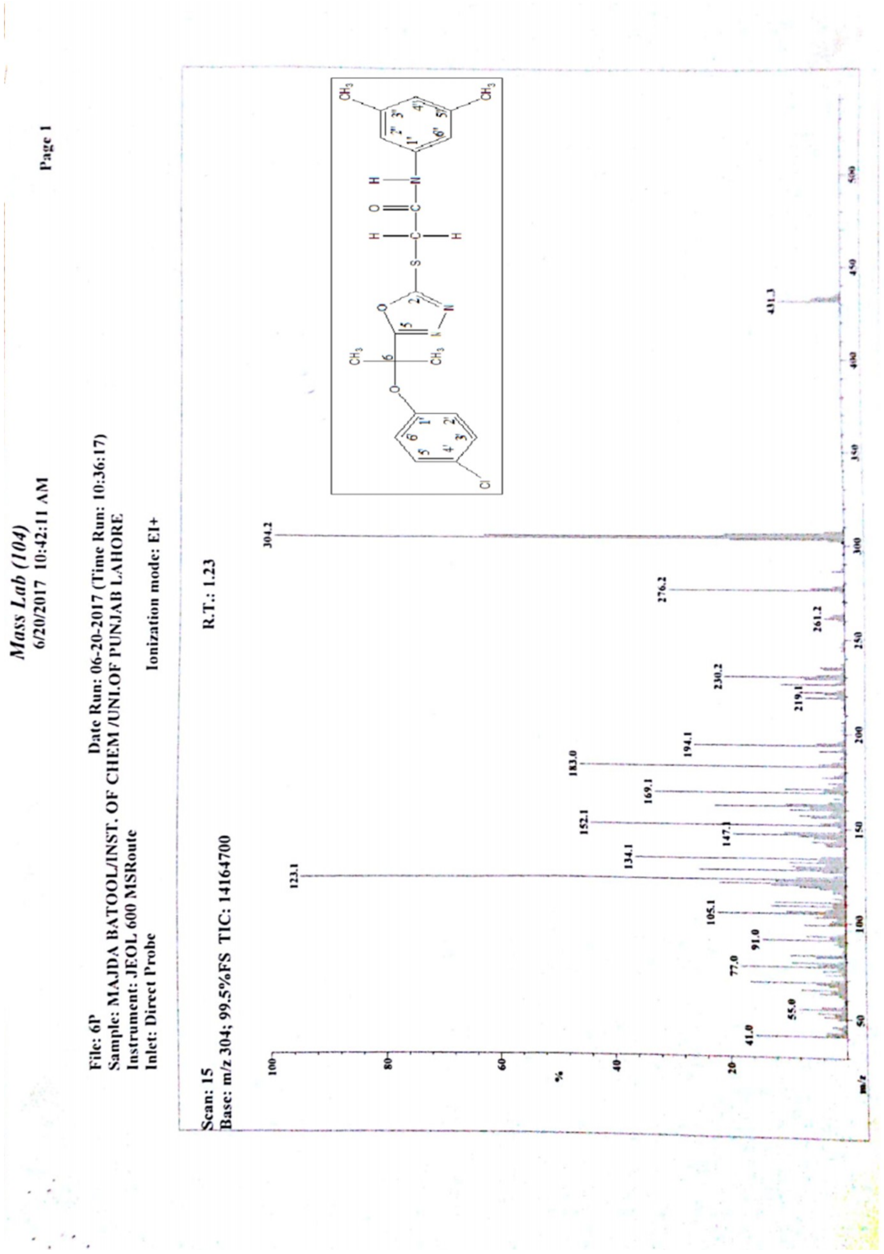

Figure S27. EI-MS of compound 3i.

Table S1. Docking results for the highest ranked biologically active ligand F-Xa.

Compd. No.	Score	*In-vivo* anticoagulant activity at 7th h (Sec)	ACE Kcal/mole	Amino acids showing hydrogen bond contacts	Distance (Å)	Amino acids showing van der Waals contacts lie within 4 Å	Amino acids showing hydrophobic contacts lie within 4 Å	Amino acids showing arene-cation contacts
**1**	3562	74	-197.87	Glu^{49}	3.22	Ser^{48}, Leu^{47}, Met^{242}, Ala^{24}, Cys^{122}, Pro^{43}, Tyr^{42}	Pro^{120}, Phe^{114}, Cys^{44}, Arg^{25}, Leu^{123}	Arg^{25}
**3a**	6270	342	-352.28	Arg^{25}	2.94	Tyr^{42}, Met^{242}, Phe^{114}, Cys^{122}, Leu^{47}, Ser^{48}, Pro^{120}, Gly^{26}, Gly^{40}, Thr^{39}	Ala^{24}, Pro^{43}	-
**3b**	5312	108	-370.05	-	-	Leu^{47}, SER^{48}, Pro^{120}, Tyr^{42}, Phe^{114}, Pro^{41}, Gly^{40}, Pro^{124}, Glu^{124}	Cys^{44}, Pro^{43}, Gly^{26}, Pro^{124}, Met^{242}, Phe^{11}, Ala^{24}, Leu^{123}	Arg^{25}
**3c**	5324	95	-253.57	Arg^{25}	2.83	Arg^{125}, Glu^{124}, Pro^{124}, Ala^{24}, Cys^{122}, Phe^{114}, Glu^{49}, Ser^{48}, Leu^{47}, Met^{242}, Asp^{239}	Leu^{123}, Arg^{25}, Pro^{120}, Leu^{235}, Lys^{236}	-
**3d**	5646	132	-287.52	Arg^{25}, Leu^{123}	3.19, 3.25	-	Cys^{44}, Pro^{120}, Arg^{25}, Leu^{235}, Lys^{236}	-
**3e**	5658	167	-189.68	Arg^{25}, Leu^{123}, Pro^{124}	2.98, 3.03, 3.17	Arg^{125}, Glu^{124}, Phe^{114}, Glu^{44}, Ser^{48}	Lys^{236}, Ala^{24}, Arg^{25}, Leu^{123}, Cys^{44}	-
**3f**	5626	84	-340.54	Arg^{25}, Arg^{25}	2.99, 274	Ala^{24}, Gly^{26}, Phe^{114}, Gly^{40}, Pro^{43}, Tyr^{42}, Leu^{47}, Met^{242}, Glu^{124}	Phe^{11}, Leu^{123}, Leu^{235}, Cys^{44}, Pro^{41}, Pro^{120}	-
**3g**	5442	92	-262.48	-	-	Asp^{239}, Pro^{124}, Glu^{124}, Trp^{127}, Cys^{122}	Ala^{24}, Phe^{11}, Cys^{44}, Arg^{25}, Lys^{236}, Arg^{125}, Leu^{235}	-
**3h**	5612	130	-311.48	-	-	Thr^{39}, Gly^{40}, Pro^{41}, Gly^{26}, Pro^{120}, Cys^{44}, Leu^{47}, Asp^{239}, Ala^{24}, Phe^{11}, Glu^{124}	Pro^{43}, Leu^{123}, Phe^{114}	Arg^{25}

**3i**	5662	214	-312.12	-	-	Pro^{41}, Tyr^{42}, Pro^{120}, Leu^{47}, Cys^{44}, Met^{242}, Ala^{24}, Asp^{239}, Gly^{26}, Pro^{43}, Thr^{39}, Gly^{40}	Arg^{25}	Arg^{25}
**RPR200095**	5192	110	-197.81	-	-	Leu^{47}, Ser^{48}, Cys^{23}, Tyr^{51}, Cys^{122}, Gly^{26}, Ala^{24}, Phe^{11}, Pro^{124}, Leu^{235}, Asp^{239}, Leu^{235}	Lys^{243}, Arg^{25}, Met^{242}	-

No. = Specific code assigned to ligand; ACE= Atomic contact energy calculated by *Patch Dock* (kcal/mol); Distance = hydrogen bond length calculated from docked pose by using *Ligand interaction* tool of *Patch Dock.*

Table S2. DFT computed molecular properties (all in eV, except dipole moment which is in the units of Debye) for oxadiazoles derivatives obtained at B3LYP/6-31G** level of theory.

**Sr. No.**	**Compound name**	E_{HOMO}	E_{LUMO}	E_{gap}	**Ionisation potential (I)**	**Electron affinity (A)**	**Chemical hardness (η)**	**Chemical softness (S)**	**Chemical potential (μ)**	**Electronegativity (χ)**	**Electrophilicity (ω)**	**Dipole moment (D)**
1	**1**	-6.60	-0.58	6.02	6.60	0.58	3.01	0.17	-3.59	3.59	2.14	3.66
2	**3a**	-6.25	-0.64	5.61	6.25	0.64	2.80	0.18	-3.44	3.44	2.12	1.84
3	**3b**	-6.14	-0.64	5.51	6.14	0.64	2.75	0.18	-3.39	3.39	2.09	1.83
4	**3c**	-6.15	-0.60	5.54	6.15	0.60	2.77	0.18	-3.37	3.37	2.06	2.20
5	**3d**	-6.05	-0.57	5.48	6.05	0.57	2.74	0.18	-3.31	3.31	1.99	2.39
6	**3e**	-6.07	-0.58	5.49	6.07	0.58	2.75	0.18	-3.32	3.32	2.01	2.19
7	**3f**	-5.95	-0.57	5.39	5.95	0.57	2.69	0.19	-3.26	3.26	1.97	2.36
8	**3g**	-6.41	-0.54	5.87	6.41	0.54	2.94	0.17	-3.47	3.47	2.05	2.14
9	**3h**	-5.97	-0.53	5.43	5.97	0.53	2.72	0.18	-3.25	3.25	1.94	2.67
10	**3i**	-6.08	-0.54	5.54	6.08	0.54	2.77	0.18	-3.31	3.31	1.98	2.37

© 2018 by the authors. Submitted for possible open access publication under the terms and conditions of the Creative Commons Attribution (CC BY) license (http://creativecommons.org/licenses/by/4.0/).
